# Dual-target electrochemical aptasensor based on Mil101(Fe)-CQD-TiO_2_ ternary composite for *Cryptosporidium* and cadmium ion detection

**DOI:** 10.1007/s00604-025-07567-2

**Published:** 2025-10-02

**Authors:** Indiphile Nompetsheni, Nithyadharsen Palaniyandy, Ntuthuko Wonderboy Hlongwa, Xolile Fuku

**Affiliations:** Institute for Nanotechnology and Water Sustainability (iNanoWS), College of Science, Engineering and Technology, University of South Africa, Roodepoort, 1709 FL South Africa

**Keywords:** Aptasensor, Modified glassy carbon electrode, Square wave voltammetry, Mil101(Fe)-CQD-TiO_2_ ternary composite, *Cryptosporidium*, Cadmium, Water pollution

## Abstract

A Mil101 (Fe)-carbon quantum dot-titanium dioxide (Mil101(Fe)-CQD-TiO_2_) ternary composite was prepared using the hydrothermal method. The composite was thoroughly characterized using various physicochemical techniques to investigate the crystallinity of the materials, the functional groups present, chemical vibrations, elemental composition, and morphology, confirming the formation of the ternary composite. The electrochemical properties of the ternary composite were then evaluated through cyclic voltammetry (CV) and electrochemical impedance spectroscopy (EIS). This ternary composite served as an electrode modifier for aptasensor fabrication. The aptasensor exhibited an electroactive surface area of 3.22 cm^2^, indicating a higher number of active sites for analyte interaction. The aptasensing platform was optimized and evaluated in phosphate buffer solution and real water samples using square wave voltammetry (SWV). Additionally, the sensor demonstrated a limit of detection (LOD) of 0.001 µM for *Cryptosporidium* and 0.073 µM for cadmium ions with high sensitivities of 9 × 10^−4^ mA/µM and 0.127 mA/µM in buffer solutions. The aptasensor demonstrated excellent selectivity, with recoveries ranging from 67 to 98.1% for *Cryptosporidium* and 72 to 89% for Cd^2+^.

**Graphical Abstract:**

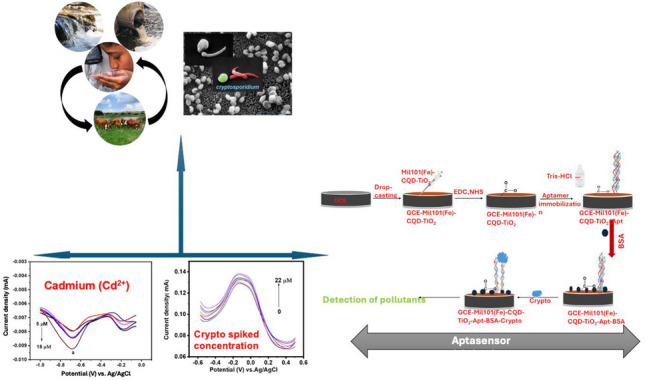

## Introduction

Over the past three decades, the World Health Organization (WHO) has reported growing global concerns about the effects of environmental pollution on public health, particularly the increasing prevalence of water contamination from pathogens and heavy metals. *Cryptosporidium* is a protozoan parasite that has emerged as a significant cause of diarrhoeal illness [1]. This parasite can be transmitted via an oral-fecal route, and its oocysts are resistant to chlorination, one of the standard disinfecting methods [1, 2]. The presence of *Cryptosporidium* in water bodies can lead to gastrointestinal diseases in both animals and human beings [3, 4]. Additionally, *Cryptosporidium* poses a significant threat to young children and immunocompromised patients, particularly patients with HIV and AIDS, as well as healthy adults [5, 6]. On the other hand, cadmium ions (Cd^2+^) are among the most toxic and hazardous metals found in environmental systems[7]. Consuming water contaminated with Cd^2+^ ions can pose detrimental health risks, including cancer, diabetes, organ injuries, and heart problems [8, 9]. To address this problem, water quality standards have established a maximum acceptable concentration of Cd^2+^ ions in water bodies to be 0.003 mg/L [10]. Both *Cryptosporidium* and cadmium ions have negative effects on animals, humans, and aquatic species [11]. To monitor and detect cadmium ions and *Cryptosporidium* in water bodies, several spectroscopic techniques have been developed. These methods include UV–Vis spectroscopy, surface-enhanced spectroscopy, inductively coupled plasma mass spectroscopy, and electrochemical methods [12, 13]. Among these methods, electrochemical methods, particularly biosensors, have demonstrated high efficiency in quantifying and monitoring both pathogens and heavy metals in water bodies [14, 15]. Biosensors are known for their advantages, including portability, suitability for on-site analysis, efficiency, user-friendliness, selectivity, stability, sensitivity, and demonstrating low limits of detection [16–19].

The development of biosensors includes three components: bio-recognition element (such as aptamers, DNA, enzymes), nanomaterial, and detector [20, 21]. Aptamers have proven effective in detecting various pollutants at a trace level [22, 23]. One of the essential aspects of developing an aptasensor is the immobilization of aptamers on the surface of the electrode. Recently, nanomaterials have been employed as a vehicle for aptamer immobilization on the surface of working electrodes [24, 25]. Metal–organic frameworks (MOFs) have gained interest as electrode materials due to their unique porosity. This property facilitates electrochemically driven ion exchange, thanks to their tunable pore sizes, large surface area, accessible metal sites, and adjustable structures [26–28]. Among MOFs, Fe-based Mil101 has been reported as a suitable electrocatalyst for electrochemical sensors due to the semiconductor iron that possesses a unique d-electron configuration and different valence states (Fe^3+^/Fe^2+^) [26, 29]. The properties of metal–organic frameworks (MOFs), particularly their abundant metallic active sites, enhance electron mobility at the electrode surface [30]. For this reason, MOFs are utilized in a wide range of applications, including energy storage, sensing, clinical applications, catalysis, adsorption, and drug delivery [31, 32]. On the other hand, carbon quantum dots (CQDs) are an innovative type of carbon nanomaterial known for their remarkable characteristics [33]. These characteristics include high surface area, chemical stability, good photoluminescence, water solubility, simple synthesis methods, easy functionalization, and unique optical and electrical properties [34–38]. These features enable CQD to have a wide range of applications, including in biosensing [39]. Metal oxides improve the electrochemical properties of carbon nanostructured materials [40]. Metal oxide–based nanocomposites are widely used in various electrochemical applications, including solar cells, supercapacitors, sensing, immunosensors, and antimicrobial analysis [41, 42]. Titanium dioxide (TiO_2_) is one of the most used metal oxides in electrochemical sensing due to its excellent electrical properties, biocompatibility, stability, higher surface area-to-volume ratio, and non-toxicity [43, 44].

In this study, we propose the use of Mil101(Fe)-carbon quantum dot-titanium ternary composite as an electrode modifier for aptasensor development. The high surface area of the ternary composite facilitates effective interaction with the analyte, enabling the aptasensor to detect the analyte at a trace level [45, 46]. Additionally, the conductivity of the ternary composite enhances the sensitivity and selectivity of the aptasensor, resulting in increased accuracy [47]. In a study conducted by Fan et al. [48], a sensor was fabricated using Mil101(Fe)-CQD composited to detect Fe^3+^ and Cu^2+^ ions in water. The sensor achieved detection limits of 2.3 ppb and 1.3 ppb. Another study by Abedi et al. [49] developed an aptasensor based on Mil101-CNTs for the detection of *Pseudomonas Aeruginosa* bacteria, which exhibited a LOD of 1.0 CFU/mL. Additionally, Abdelghany et al. [50] designed a carbonized-metallic framework sensor for the rapid detection of Cd^2+^, Cu, and Pb^2+^ in water bodies. The sensor demonstrated sensitivity to all metals, with limits of detection of 0.0154 ppm, 0.00448 ppm, and 0.016 ppm. Karimi-Maleh and colleagues developed an electrochemical sensor using a Co_3_O_4_@MOF nanocomposite to detect the organophosphorus insecticide fenamiphos. This sensor achieved a detection limit of 3.0 × 10^−12^ M [51]. In another study, Yesurajan and his team investigated cytokine detection using a Ni-Cu-MOF-based immunosensor, which demonstrated an impressive detection limit of 2.0 fg/mL [52].

## Experimental work

### Chemicals and methods

Terephthalic acid (H_2_BDC), hydrochloric acid (HCl,60%), N’N dimethylformamide (DMF), absolute ethanol, bovine serum albumin (BSA), titanium isopropyl-oxide, alumina polishing powders (0.1, 0.3, and 0.05 µM), N-hydroxy succinimide (NHS,98%), tris-hydrochloric binding buffer, sodium hypochlorite, 1-ethyl-3,3-dimethyl-aminopropyl carbodiimide hydrochloride (EDC,98%), sulfuric acid (H_2_SO_4_,98%), and lemon peels were sourced from local produce store in Florida. The Crypto primer used was CTC TGA CTG TAA CCA CGG TGG TCC CGC AAA ATG CAC GAC GAG, the aptamer sequence used was (R4-6): HS-CH_3_CH_2_−5′-CTC CTTGA CTG TAA CCA CGG TGG TCC CGC AAA ATG CAC GAC GAG TCT TGC TTC TGA TCT GCA TAG GTA GTC CAG AAG CC-3′, and the mismatched analyte used was TGC TTC TGA TCT GCA TAG TCC AGA AGCC. Inqaba Biotechnical Industries (PTY) Ltd., located in Pretoria, South Africa, produced the synthetic primers. A stock solution (100 µM) of oligonucleotides was prepared using 0.1 M phosphate buffer solution, pH 7.2, and stored at − 20 °C when not in use.

### Synthesis of Mil101(Fe)

Mil101(Fe) was synthesized using acid mine drainage (AMD)–derived Fe (OH)_3_. 5.2 mmol of AMD-derived Fe (OH)_3_ was dissolved in HCl and heated at low temperatures [53]. This Fe solution was then combined with a dimethylformamide solution containing terephthalic acid derived from PET (H_2_BDC). The combined solution was transferred into a reactor and allowed to react for 20 h at 110 °C. Once the reaction time was complete, the solution was centrifuged and washed several times. The resulting product was dried overnight in an oven. The temperatures and durations used in this synthesis adhered to the methodologies outlined in the reported literature.

### Synthesis of carbon quantum dot

Lemon peels were used as a carbon precursor for the synthesis of carbon quantum dot (CQD) [54]. The synthesis started by washing the peels with deionized (DI) water several times in a beaker, and they were dried at 60 °C in an oven. The dried peels were crushed using a motor and a pestle. About 10 g of the lemon peel powder was combined with 100 mL of 0.1 M H_2_SO_4_. After 15 min, the peel solution was rinsed several times with 15 mL of DI water, followed by filtration. The resultant product was dried to remove all the solvents that may have been trapped. The dried product was then combined with 200 mL of sodium hypochlorite solution and stored at 25 °C for 4 h. The pH of the sample was then adjusted to 7, and the solution was transferred into a hydrothermal reactor, where it was heated for 12 h at 180 °C. The solution was then centrifuged at 4500 rpm and washed five times with DI water to remove all the unreacted organic molecules. The collected product was dried overnight in an oven at 90 °C. The temperatures and durations used in carbon quantum dots were varied between 160 °C, 180 °C, and 200 °C, with 180 °C yielding the best results.

### Synthesis of TiO_*2*_

Titanium dioxide (TiO_2_) nanoparticles were synthesized by combining 100 mL of absolute ethanol with 6 mL of titanium isopropoxide [55]. The mixture was then agitated at moderate humidity for 30 min. About 4 mL of DI water was then added to the mixture and stirred for 5 min, resulting in a white solution. Additionally, 25 mL of DI water was added to the solution. The white solution was transferred into a 200 mL reactor, and the solution underwent hydrothermal treatment for 14 h at 200 °C. The solution was washed with DI water three times using a centrifuge. The oven was utilized to dry the product overnight at 100 °C. The material was calcined in a furnace for 4 h at 450 °C. The temperature used in the synthesis of TiO_2_ varied between 180 and 200 °C, with 200 °C producing better results than 180 °C.

### Synthesis of Mil101(Fe)-CQD-TiO_2_ ternary composite

A total of 0.5 g of the synthesized Mil101(Fe) was dispersed in 150 mL of DI water. About 2 g of the synthesized CQD-TiO_2_ composite was added to the mixture, which was then sonicated for 2.5 h [56]. The solution was then transferred into a Teflon container and heated at 180 °C for 12 h. The resulting solution was centrifuged and washed three times using ethanol. The product was dried overnight in an oven. The schematic synthesis is shown in Schematic 1. The temperatures, chosen ratio, and durations used in this synthesis adhered to the methodologies outlined in the literature and were used optimally [56].

**Schematic 1:**
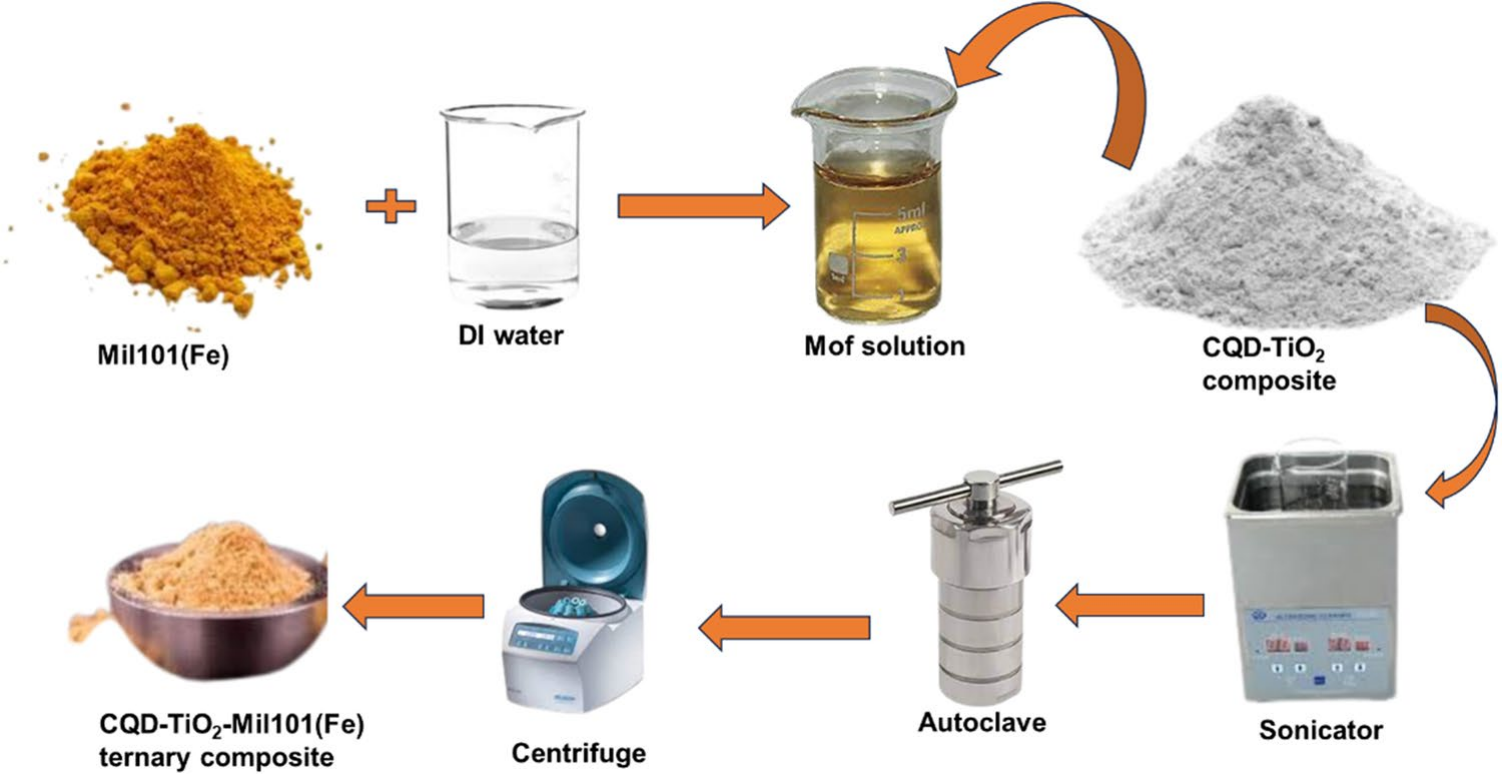
The schematic representation of Mil101(Fe)-CQD-TiO_2_ synthesis

### Fabrication of the aptasensor

The working electrodes (glassy carbon electrode (GCE)) were cleaned using alumina slurries with different roughnesses (1 µM, 0.3 µM, and 0.05 µM). After each cleaning step, the electrodes were rinsed with DI water and subjected to ultrasonication for 15 min with water and absolute ethanol to remove all the residual suspensions. About 5 mg of the synthesized Mil101(Fe)-CQD-TiO_2_ ternary composite was dispersed in 2 mL of DI water and agitated for 10 min to get a homogeneous mixture. Following this, 3 µL of EDC and NHS solutions were added along with 5 µL of a Tris–HCl binding buffer. After mixing, 10 µL of the aptamer solution was also added to the sensor solution, followed by stirring for 10 min. The cleaned GCE was then incubated in the sensor solution for 3 h at 4 °C. After the incubation period, the electrode was then rinsed with DI water and incubated in bovine serum albumin to block all the remaining active sites on the electrode surface. The schematic diagram is illustrated in Schematic 2.

**Schematic 2:**
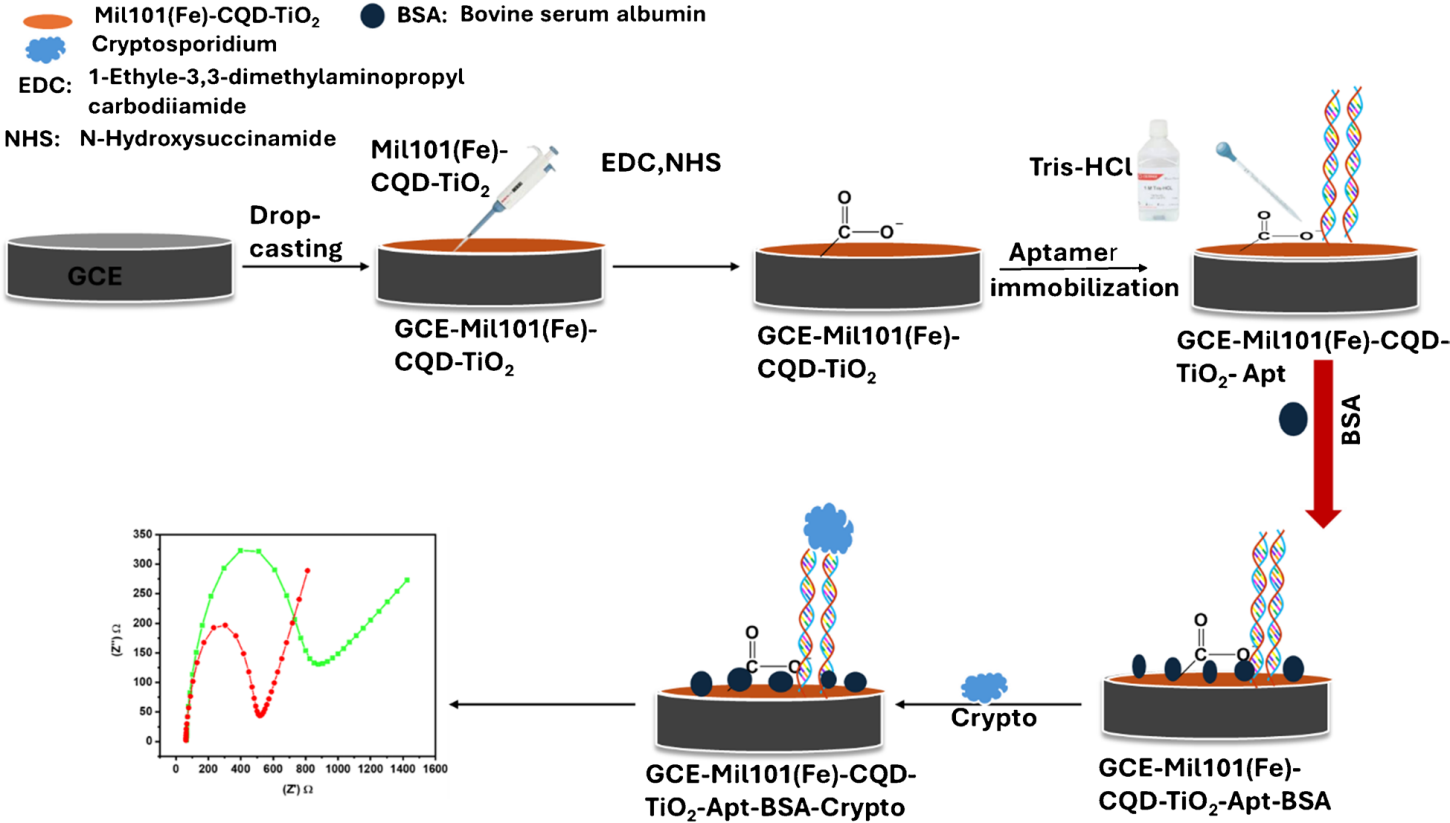
Schematic representation of the aptasensor development

#### Electrochemical experiments

The electrochemical experiments were conducted using a three-electrode system. The system included a glassy carbon electrode (GCE) as a working electrode, which has an area of 0.071 cm^2^ and a diameter of 3 mm. A silver-silver chloride (Ag/AgCl) was used as a reference, while a platinum (Pt) served as a counter electrode. All experiments were conducted using 0.1 M phosphate-buffered saline (PBS) as the electrolyte, which was purged with nitrogen (N_2_) gas. All potentials were referenced against the Ag/AgCl electrode.

#### Preparation of cadmium ion working concentrations

The working concentrations for cadmium ions were prepared by weighing 30.85 mg of cadmium nitrate, which was then dissolved in 10 mL of deionized water, resulting in a 0.01 M stock solution. From this stock solution, 100 µL was further diluted in 10 mL of deionized water to get a 0.1 mM stock solution. Different concentrations of cadmium ion, ranging from 1 to 22 µM, were prepared in 4-mL vials each using the following formula: C_1_V_1_ = C_2_V_2_.

## Instrumentation

The formation of the synthesized Mil101(Fe)-CQD-TiO_2_ ternary composite was confirmed using a variety of characterization techniques. The morphology, elemental distribution, and structure of the materials were evaluated using a JEOL JEM 2100 High-Resolution Transmission Electron Microscope (HR-TEM) operating at 200 kV. The crystallinity of the materials was analyzed using Cu Ka radiation and an XRD (Rigaku Smart Lab X-ray Diffractometer). Additionally, the chemical vibrations of the materials were assessed using a Raman microscope (WITec alpha 300 confocal), which utilized a 532 nm laser at 3 mW, coupled with Raman spectroscopy and a × 50 0.75 NA objective. Furthermore, the functional groups and elemental composition were studied using a Frontier FTIR spectrometer (PerkinElmer Highlight 400). The electrochemical analyses of the modified electrodes were investigated using various techniques, including CV, EIS, and SWV. These tests were conducted using German Biologic Science instruments, GmbH, EC Lab V11.50 Biologic (Rodeweg 20), D-37081 Göttingen.

## Results and discussions

### FTIR analysis of Mil101(Fe), CQD, TiO_2_, and Mil101(Fe)-CQD-TiO_2_ ternary composite

**Fig. 1:**
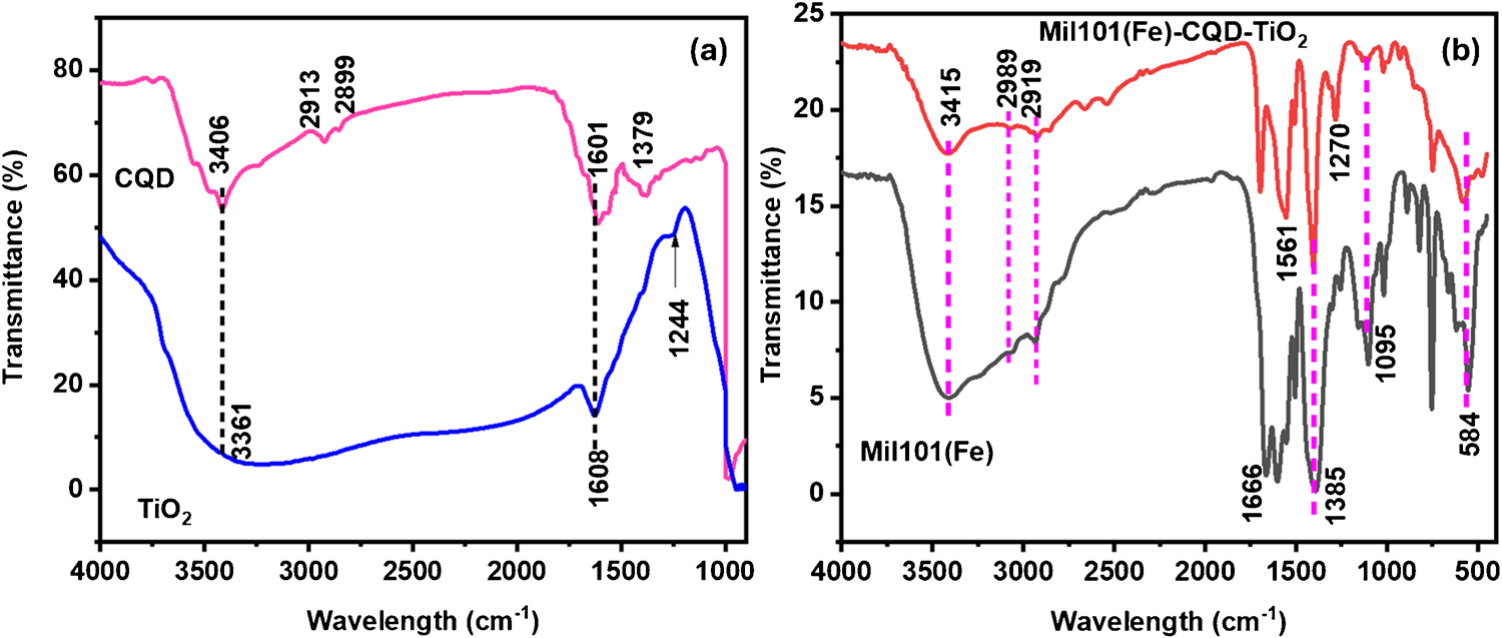
**a** The FTIR spectrum of Mil101(Fe); **b** the FTIR spectra of CQD, TiO_2_, and Mil101(Fe)-CQD-TiO_2_ ternary composite

Fourier transform-infrared (FTIR) spectroscopy is a technique that provides important insights into the properties of various nanomaterials. It is utilized to characterize chemical bonds and identify molecular structures and functional groups present within a sample [57]. The FTIR analysis was conducted over a wavelength range of 4000–400 cm^−1^ to confirm the presence of functional groups. Figure 1a,b shows the FTIR spectrum of CQD, TiO_2_, Mil101(Fe), and Mil101(Fe)-CQD-TiO_2_ ternary composite. In the FTIR spectrum of CQD, the adsorption peak observed at 1379 cm^−1^ corresponds to the formation of O–H groups, while the peak observed at 1601 cm^−1^ corresponds to the C = O from the carbonyl groups [58, 59]. The peak observed at 2913 cm^−1^ is attributed to the symmetric stretch of C-H, and the band observed at 3406 cm^−1^ corresponds to the hydroxyl bond on the surface of CQD [60]. The FTIR spectrum of TiO_2_ exhibited an absorption band at 3361 cm^−1^, which is attributed to the OH groups. At the same time, the peak observed around 1608 cm^−1^ corresponds to the C = O groups, and the peak observed at 1244 cm^−1^ corresponds to the O–H groups on the TiO_2_ surface [61–63]. These results are in quite agreement with the literature [64, 65].

The FTIR spectrum of Mil101(Fe) shows a sharp absorption peak at 584 cm^−1^, which is attributed to Fe–O vibration, while the peaks observed around 700 to 1095 cm^−1^ correspond to the C-H bonding, confirming an organic linker in the Mil101(Fe) structure [66, 67]. The peaks observed at 1385 cm^−1^ and 1666 cm^−1^ are attributed to the asymmetric and symmetric vibrations of carboxylic groups [68, 69]. The broad adsorption band observed at 3415 cm^−1^ is attributed to the stretching mode of hydroxyl groups [70]. These results agree with the literature [71, 72].

The FTIR spectrum of the ternary composite demonstrated a blue shift in the absorption peaks compared to the individual materials. A peak observed at 3415 cm^−1^ corresponds to the hydroxyl groups. Additionally, a peak associated with carbonyl groups shifted to a lower wavelength compared to Mil101(Fe), CQD, and TiO_2_, with an absorption observed at 1696 cm^−1^. The FTIR spectrum of the composite retained the characteristic features of Mil101(Fe). This observation suggests that there is coordination bonding or electronic interaction between Mil101(Fe), CQD, and TiO_2_. No new peaks were observed, indicating the successful synthesis of the Mil101(Fe)-CQD-TiO_2_ ternary composite.

### Raman analysis of Mil101(Fe), CQD, TiO_2_, and Mil101(Fe)-CQD-TiO_2_ ternary composite

**Fig. 2:**
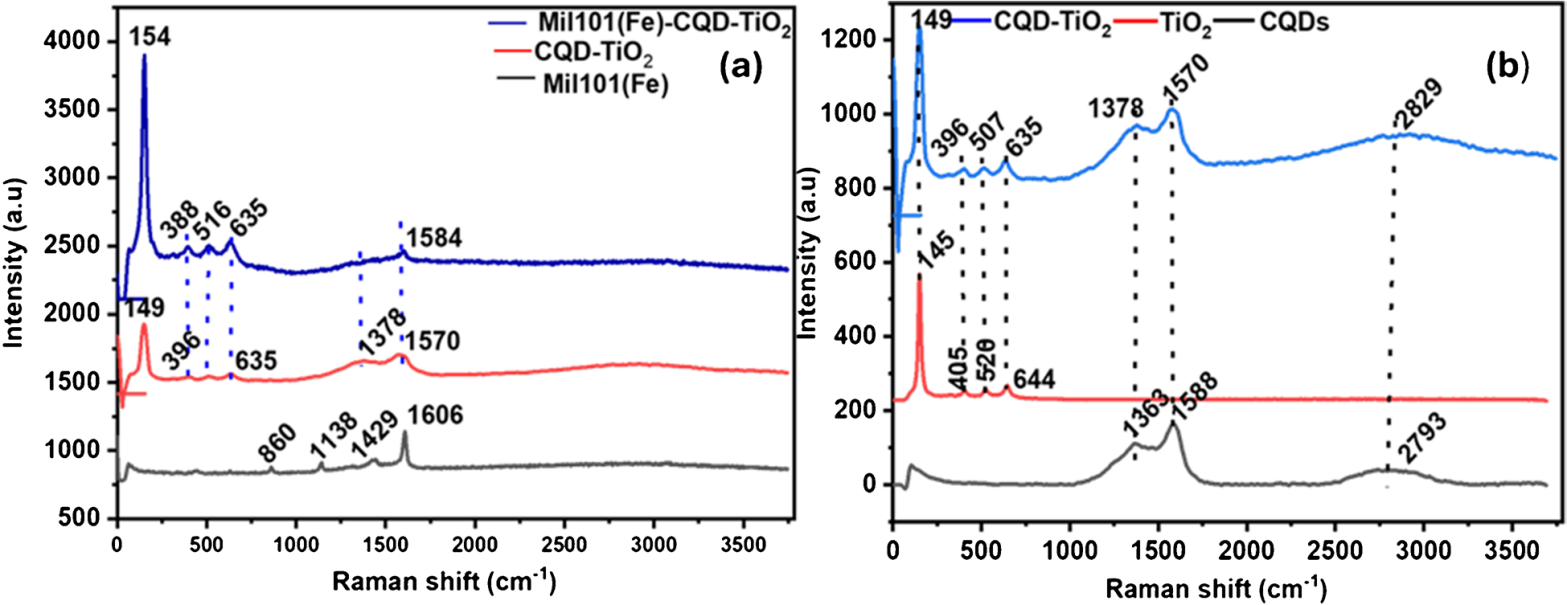
**a** The Raman spectra of Mil101(Fe)-CQD-TiO_2_ ternary composite and Mil101(Fe) and **b** the spectra for CQD and TiO_2_

**Fig. 3:**
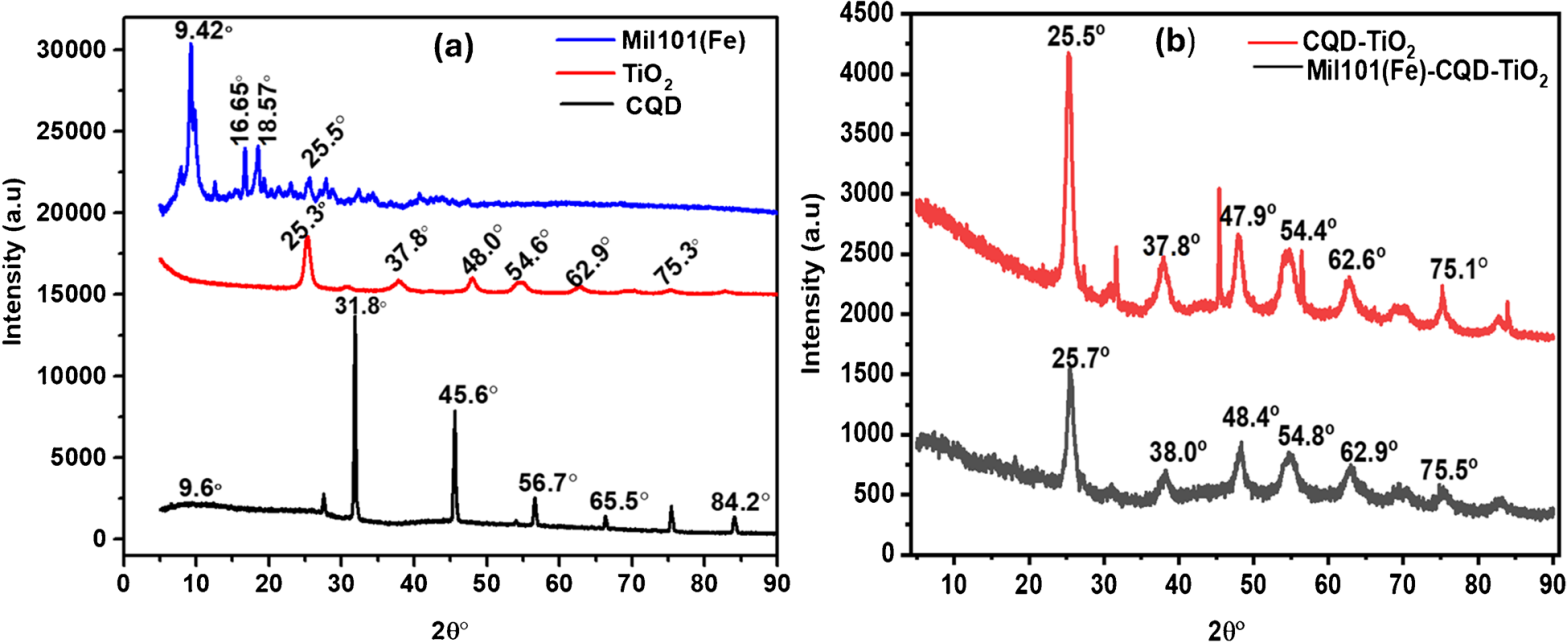
**a** The HR-TEM image of CQD, **b** TiO_2_, **c** enhanced image of (**b**), **d** Mil101(Fe), **e** Mil101(Fe)-CQD-TiO_2,_ and **f** the interplanar spacing for Mil101(Fe)-CQD-TiO_2_ ternary composite

Raman spectroscopy is a non-invasive vibrational spectroscopic technique used for both qualitative and quantitative purposes. Quantitative analysis involves measuring the intensity of the scattered radiation, while qualitative analysis detects the frequency of scattered radiation. Raman spectroscopy has become essential for material characterization, enabling the examination of vibrational modes and the nature of chemical bonds within a sample. In this work, Raman spectroscopy was employed to investigate the structural and vibrational modes of Mil101(Fe), CQD, TiO_2_, and the Mil101(Fe)-CQD-TiO_2_ ternary composite, as shown in Fig. 2. The Raman spectra of Mil101(Fe) showed four distinct Raman bands, which are related to the organic ligands within the Mil101(Fe) structure, as illustrated in Fig. 3a. The band observed at 860 cm^−1^ corresponds to the C-H vibrations, while the band at 1138 cm^−1^ is associated with the C–C bond in the benzene ring [73, 74]. The bands observed at 1429 cm^−1^ and 1606 cm^−1^ are attributed to the C = O stretching vibration in the carboxylic group [75]. The Raman spectra of CQD displayed three Raman bands, as shown in Fig. 3b. The D and G of CQD appeared at 1363 cm^−1^ and 1588 cm^−1^, and the peak observed at 2793 cm^−1^ confirms the amorphous nature of the synthesized CQD. These results are consistent with the XRD analysis of CQD. The D band corresponds to the vibration of sp^2^ carbon atoms, whereas the G band is associated with the disordered carbon structures.

The TiO_2_ Raman spectra showed four distinct Raman bands as illustrated in Fig. 3b. The peak at 644 cm^−1^ is associated with E_g_, whereas the band observed at 520 cm^−1^ corresponds to the A_1g_. Additionally, the bands observed at 405 cm^−1^ and 145 cm^−1^ correspond to B_1g_ and E_g_. The vibrational frequencies confirmed the presence of the anatase phase of titanium dioxide (TiO_2_). These results are consistent with XRD and HR-TEM analyses. The Raman spectrum of the ternary composite showed bands with increased intensities, confirming the incorporation of Mil101(Fe), CQD, and TiO_2_ into the ternary composite. For instance, a band observed at 149 cm^−1^ in the TiO_2_ spectrum shifted to 154 cm^−1^ in the Mil101(Fe)-CQD-TiO_2_ ternary composite spectrum with increased intensity. Additionally, the peak appeared at 1584 cm^−1^ in the ternary composite, which was observed at 1588 cm^−1^ in the CQD Raman spectrum. The deformation of the Mil101(Fe) bands in the ternary composite indicates strong electronic interaction between the Fe centers and oxygen-containing groups from CQD and TiO_2_. These results are consistent with those observed in the HR-TEM analysis of the ternary composite.

### XRD analysis of Mil101(Fe), CQD, TiO_2_, CQD-TiO_2_, and Mil101(Fe)-CQD-TiO_2_ ternary composite

**Fig. 4:**
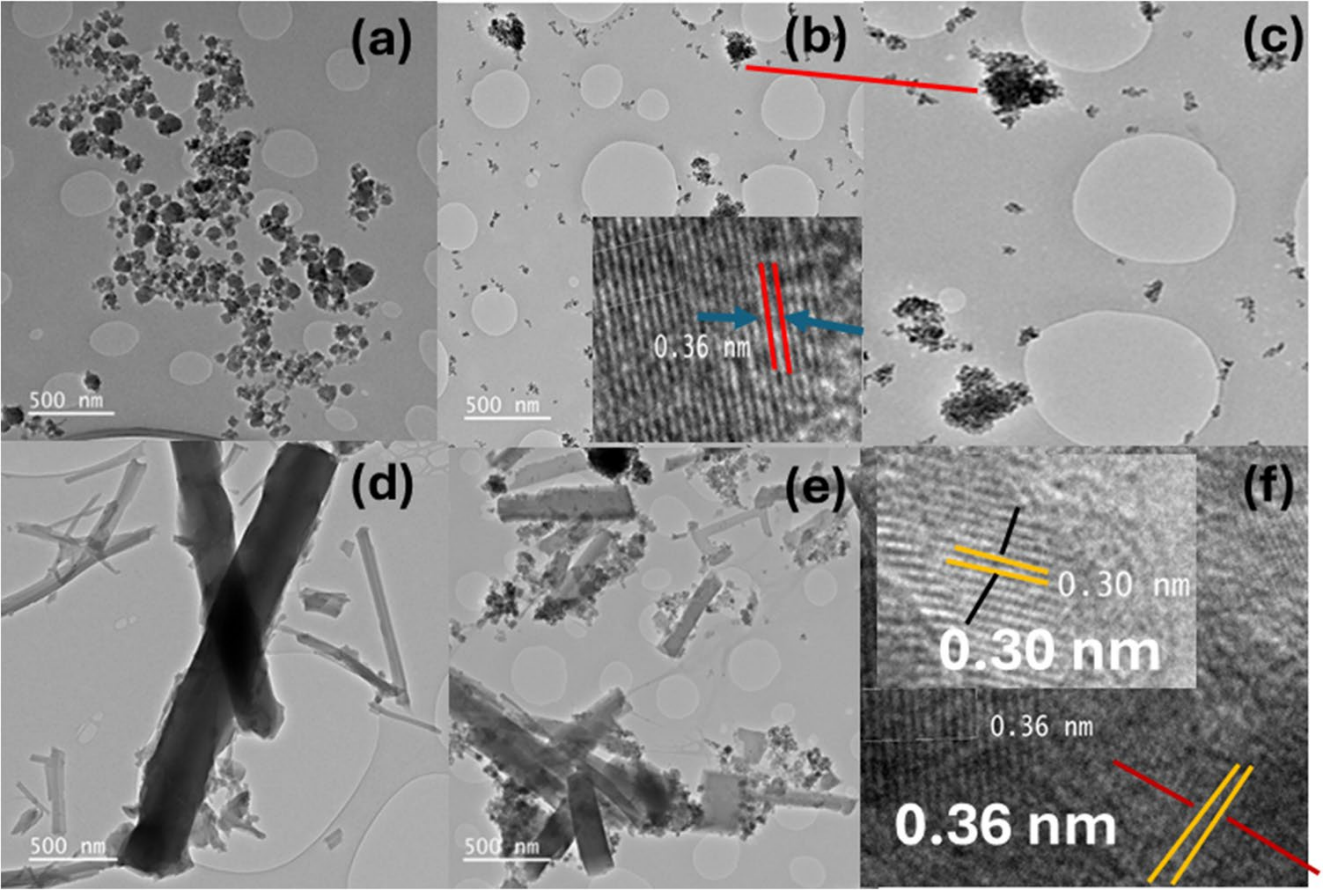
**a** The XRD of CQD, TiO_2_, and CQD-TiO_2_ and **b** the XRD of Mil101(Fe)-CQD-TiO_2_ ternary composite

X-ray diffraction (XRD) is a powerful analytical technique used to identify the crystalline structure and phase composition of materials. The method works by shining X-rays on a material and analyzing how they diffract based on the atomic arrangement within the material [76]. This provides information about the material’s crystal structure, phase composition, crystal orientation, size, and phase identification. XRD was used to evaluate the crystalline structure, *d*-spacing, and phase identification of the synthesized nanomaterials [77]. Figure 4 illustrates the XRD of CQD, TiO_2_, Mil101(Fe), and Mil101(Fe)-CQD-TiO_2_ ternary composite. CQD fails to have a periodic crystal structure [78] and is unable to scatter electrons [79]. It features an unstructured ring that exhibits an X-ray diffraction peak between 20.4 and 24.6° [80]. The broad diffraction peak observed at 2θ = 22 to 25° corresponds to the (002) reflection plane of graphite. The synthesized CQD in this study exhibited some peaks within the 2θ range 9–90°. The broad diffraction peak appeared between 9 and 20° in 2θ, suggesting the amorphous nature of CQD [81]. These results are consistent with Raman analysis. The shift in the broad diffraction peak is due to the type of precursor used, the synthesis method, and the temperatures applied during synthesis [82]. The results suggest that the carbon atoms in the carbon quantum dot are arranged in a disordered manner. This finding is consistent with the results of the Raman analysis. Additionally, the presence of small and sharp peaks in the XRD patterns of CQD indicates the presence of residual organic materials within the amorphous carbon region. Research conducted by Thulasi et al. and Imran Din et al. [82, 83] also focused on the synthesis of CQD using green chemistry, yielding results that are consistent with those found in this study.

The XRD patterns of the synthesized TiO_2_ displayed sharp, narrow, and intense peaks, indicating the crystalline nature of the TiO_2_. The patterns of TiO_2_ exhibited prominent peaks at 2θ values of 25.3°, 37.8°, 40.0°, 54.6°, 62.9°, and 75.3°, which correspond to the crystal planes (101), (0.04), (200), (105), (211), (204), and (107) [84, 85]. These results indicate that the TiO_2_ is in the anatase phase, which is consistent with the Raman analysis. The XRD patterns of the synthesized Mil101(Fe) revealed peaks at 2θ values of 9.42°, 16.65°, 18. 57°, and 25. 5°. The exhibited XRD patterns correspond to the crystal planes of (311), (111), (511), and (852) [68, 86–88]. The observed peaks confirmed the high crystallinity of Mil101(Fe), and the findings are consistent with previous studies.

Average particle size was determined by applying the Scherrer equation to the apparent full width at half maximum intensity (FWHM) of the most prominent peak, as illustrated in the equation below [52].

$$D=\frac{k\lambda }{\beta cos\theta }$$

λ is the wavelength of the X-ray beam (0.15405 nm), *K* is equal to 0.9, *D* refers to the average particle size, β is the FWHM, and θ is the Bragg angle of the intense peak. Table 1 depicts the calculated *d*-spacing and crystallite size of the synthesized nanomaterials. The *d*-spacing refers to the distance between two parallel atomic planes, which leads to diffraction peaks [89]. In contrast, crystalline size represents the coherent volume in the material corresponding to the diffraction peak [90]. The composite exhibited a crystallite size of 7.72 nm, which is lower compared to both TiO_2_ (8.10 nm) and CQD (30.8 nm), as shown in Table 1. These results suggest that combining Mil101(Fe), CQD, and TiO_2_ into a ternary composite results in a reduction in crystallite size compared to individual materials [91]. A smaller crystallite size typically leads to a high surface area-to-ratio, which enhances the interaction with analytes and is essential for effective sensing [92].

**Table 1:** The calculated crystallite sizes and *d*-spacing of Mil101(Fe), CQD, TiO_2_, and Mil101(Fe)-CQD-TiO_2_ ternary composite

Material	Crystallite size (nm)	*d*-spacing (nm)	2θ
TiO_2_	8.10	0.35	25.8°
CQD	30.8	0.28	31.1°
Mil101(Fe)	4.33	0.94	9.44°
Mil101(Fe)-CQD-TiO_2_	7.71	0.34	25.7°

### HR-TEM analysis of CQD, TiO_2_, Mil101(Fe), and Mil101(Fe)-CQD-TiO_2_ ternary composite

High-resolution transmission microscopy (HR-TEM) is a phase-contrast imaging technique that enables the acquisition of images at near-atomic resolution. This method is particularly effective for analyzing the crystallinity, lattice planes, crystal phases, and defects in nanomaterials. HR-TEM is an excellent technique for studying the elemental distribution and morphological structure of nanomaterials on the nanoscale [93, 94]. HR-TEM was used to investigate the crystallinity, lattice planes, and morphological structures of Mil101(Fe), CQD, TiO_2_, and the Mil101(Fe)-CQD-TiO_2_ ternary composite. Figure 3a–f illustrates the HR-TEM images of CQD, TiO_2_, Mil101(Fe), and Mil101(Fe)-CQD-TiO_2_ ternary composite. Figure 3a shows monodispersed CQD particles with a spherical morphology. These findings align with the reports by Gemachu and Bogale and Hassanzadeh-Tabrizi [91, 92].

Figure 3b,c illustrates the HR-TEM image of the synthesized TiO_2_ NPs. The results indicate that the structure of the prepared TiO_2_ NPs is agglomerated and has an irregular morphology. The agglomeration occurs because particles are unstable in the nanoparticle form, and they tend to clump together until they achieve stability [95]. These results are consistent with the existing literature [96]. The TiO_2_ NPs exhibited a circular-like shape with a uniform structure. Additionally, the circular form of TiO_2_ allows for good dispersion in various solutions, offering a vast surface area that enhances interaction with other materials [97, 98]. The irregular and agglomerated structure of TiO_2_ can decrease the number of active sites on its surface, which in turn reduces the effective surface area and hampers the performance of the composite. Additionally, TiO_2_ exhibited a lattice fringe with an interspacing of 0.36 nm. This interspacing corresponds to the (101) crystal plane of anatase, indicating that TiO_2_ has a crystalline structure [84]. This observation is consistent with the XRD results. The HR-TEM of Mil101(Fe) displayed a large rod-like or needle-shaped morphology with a smooth surface, as illustrated in Fig. 3d. Some of the rod-like structures overlapped, indicating an aggregation of Mil101(Fe) particles. The observed characteristics of Mil101(Fe) suggest anisotropic growth during the hydrothermal synthesis, influenced by the temperature [99, 100].

Figure 3e shows the HR-TEM image of the synthesized Mil101(Fe)-CQD-TiO_2_ ternary composite. The image revealed well-distributed arrangements of CQD and TiO_2_ on the surface of Mil101(Fe). The ternary composite exhibited lattice fringes with intercellular spacings of 0.30 nm and 0.36 nm, confirming the crystallinity of the composite. The interplanar spacings of 0.36 nm and 0.30 nm exhibited by the ternary composite confirm the presence of TiO_2_ in the composite, corresponding to the 101 crystal phase of TiO_2_. The results are consistent with the XRD analysis.

### EDS analysis of Mil101(Fe), CQD, TiO_2_, and Mil101(Fe)-CQD-TiO_2_ ternary composite

**Fig. 5:**
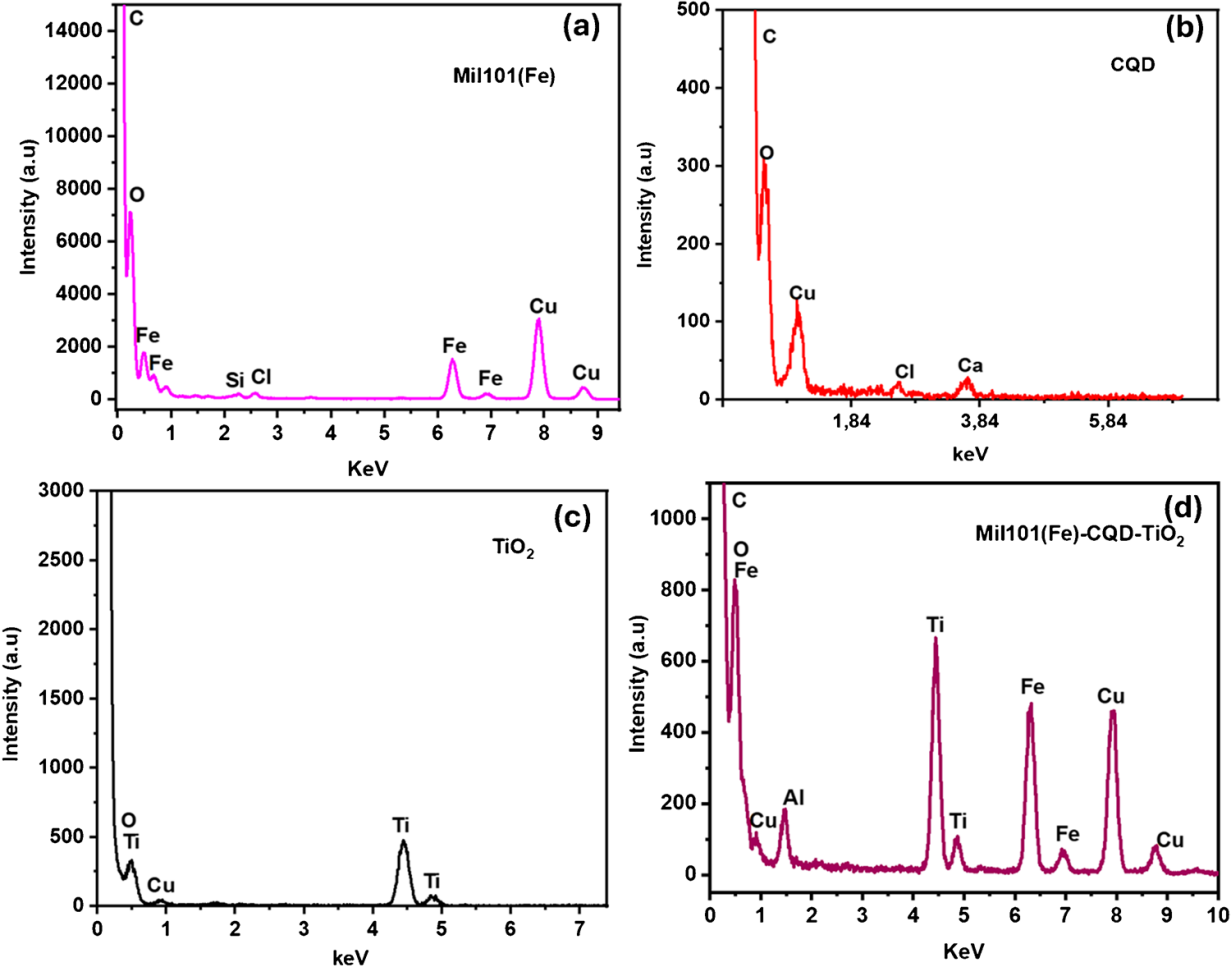
The EDS spectra of **a** Mil101(Fe), **b** CQD, **c** TiO_2_, and **d** Mil101(Fe)-CQD-TiO_2_ ternary composite

Energy dispersive spectroscopy (EDS) is a technique used to quantify and identify the components of a sample [101]. EDS measures the intensity or energy of X-rays emitted from a sample when it is exposed to the electron beam. Additionally, it is also used in conjunction with scanning electron microscopy or transmission electron microscopy to create elemental mapping of the sample [101]. Elemental mapping allows researchers to see the distribution of elements within a sample. In this work, EDS was used to identify the elements present in Mil101(Fe), CQD, and TiO_2_ and to confirm the formation of the Mil101(Fe)-CQD-TiO_2_ ternary composite. Figure 5a–d shows the EDS spectra of different samples. Figure 5a illustrates the EDS spectrum of Mil101(Fe), which primarily consists of iron (Fe), oxygen (O), and carbon (C). This composition is aligned with FTIR analysis, confirming the presence of hydroxyl, carbonyl, and Fe metal on the surface of Mil101(Fe). Additionally, the presence of the chlorine peak, copper, and silicon is due to the impurities present in the sample.

Figure 5b shows the EDS spectrum of CQD, which is composed of carbon and oxygen, confirming the presence of oxygen-containing groups on the surface of the carbon quantum dot. The peaks observed for copper (Cu), calcium (Ca), and chlorine are attributed to the impurities in CQD. In Fig. 5c, the elemental composition of titanium dioxide is illustrated. The TiO_2_ spectrum primarily exhibited titanium and oxygen, and the peak observed for Cu is also attributed to impurities in the titanium sample. The EDS spectrum exhibited peaks for oxygen, iron, and titanium, confirming the successful synthesis of the ternary composite as shown in Fig. 5d. The Cu and aluminium peaks are associated with impurities present in the sample.

**Fig. 6:**
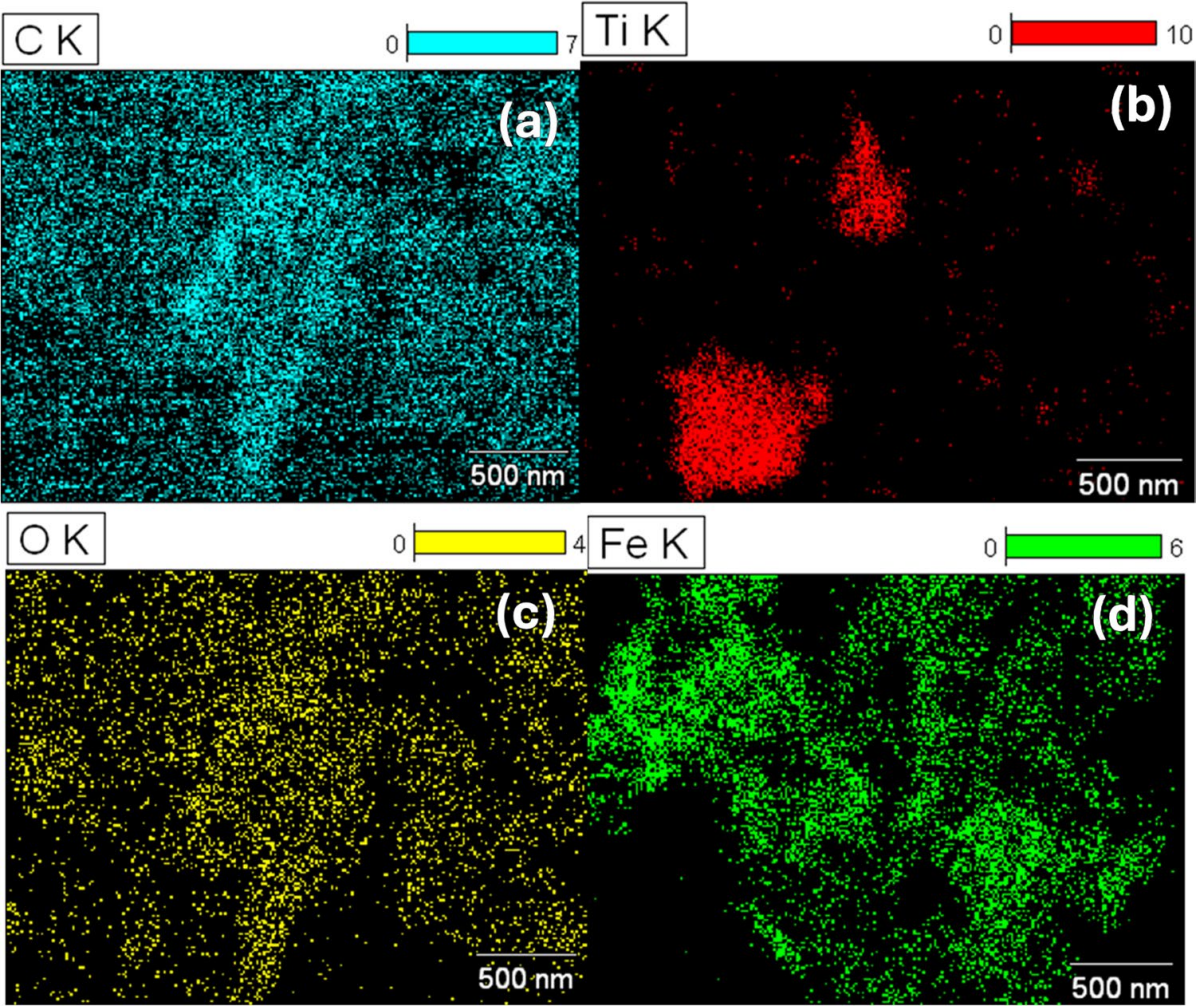
The elemental mapping of **a** carbon, **b** titanium, **c** oxygen, and **d** iron

The elemental mapping of the materials is illustrated in Fig. 6a–d. Elemental mapping helps to understand the distribution of different elements within the synthesized materials. Figure 6a–d displays the elemental maps for carbon, titanium, oxygen, and iron. The mapping results are consistent with the EDS spectra of the synthesized materials, confirming the presence of all the elements indicated by the different spectra. Table 2 summarizes the results observed in the EDS spectra, displaying the atomic weight percentages of each element present in the synthesized materials. For instance, the Mil101(Fe)-CQD-TiO_2_ ternary composite sample revealed the presence of titanium (Ti), oxygen (O), and iron (Fe) elements, confirming the incorporation of carbon quantum dots, titanium dioxide, and Mil101(Fe).

**Table 2:** The atomic %weight of CQD, TiO_2_, Mil101(Fe), and Mil101(Fe)-CQD-TiO_2_ ternary composite

Material	Element	Weight. %	Error	Atomic %	Error %	Formula
**CQD nanomaterial**	O K	98.10	± 2.57	99.27	2.61	O
	C K	0.00	± 0.00	0.00	0.00	C
	Cu K	0.06	± 0.00	0.00	0.00	Cu
	Ca K	0.41	± 0.06	0.17	0.03	Ca
	Cl K	0.89	± 0.16	0.41	0.07	Cl
	Total	100		100		
**TiO**_**2**_** nanomaterial**	O K	96.28	± 1.11	98.76	1.14	O
	Ti K	3.27	± 0.03	1.12	0.01	Ti
	Cu K	0.06	± 0.00	0.00	0.00	Cu
	Total	100		100		
**Mil101(Fe)**	O K	0.00	± 0.00	0.00	0.00	O
	C K	0.00	± 0.00	0.00	0.00	C
	Cl K	40.51	± 1.03	48.85	1.24	Cl
	Fe K	33.20	± 0.42	25.42	0.32	Fe
	Cu K	15.64	± 0.53	10.52	0.36	Cu
	Si K	5.31	± 0.66	8.09	1.00	Si
	S k	5.34	± 0.62	7.12	0.83	S
	Total	100		100		
**Mil101(Fe)-CQD-TiO**_**2**_	O K	94.12	± 1.73	97.05	1.78	O
	Cu K	0.46	± 0.01	0.12	0.00	Cu
	Ti K	1.16	± 0.03	0.40	0.01	Ti
	Al K	3.70	± 0.03	0.14	2.26	Al
	Fe K	0.56	± 0.01	0.017	0.00	Fe
	Total	100		100		

## Electrochemical analysis (CV and EIS)

CV is a powerful electrochemical technique used to investigate oxidation–reduction processes of molecular species. CV also examines the kinetic reactions occurring at the electrode–electrolyte interface, including electron transfer, absorption processes, and diffusion-controlled processes [102].

**Fig. 7:**
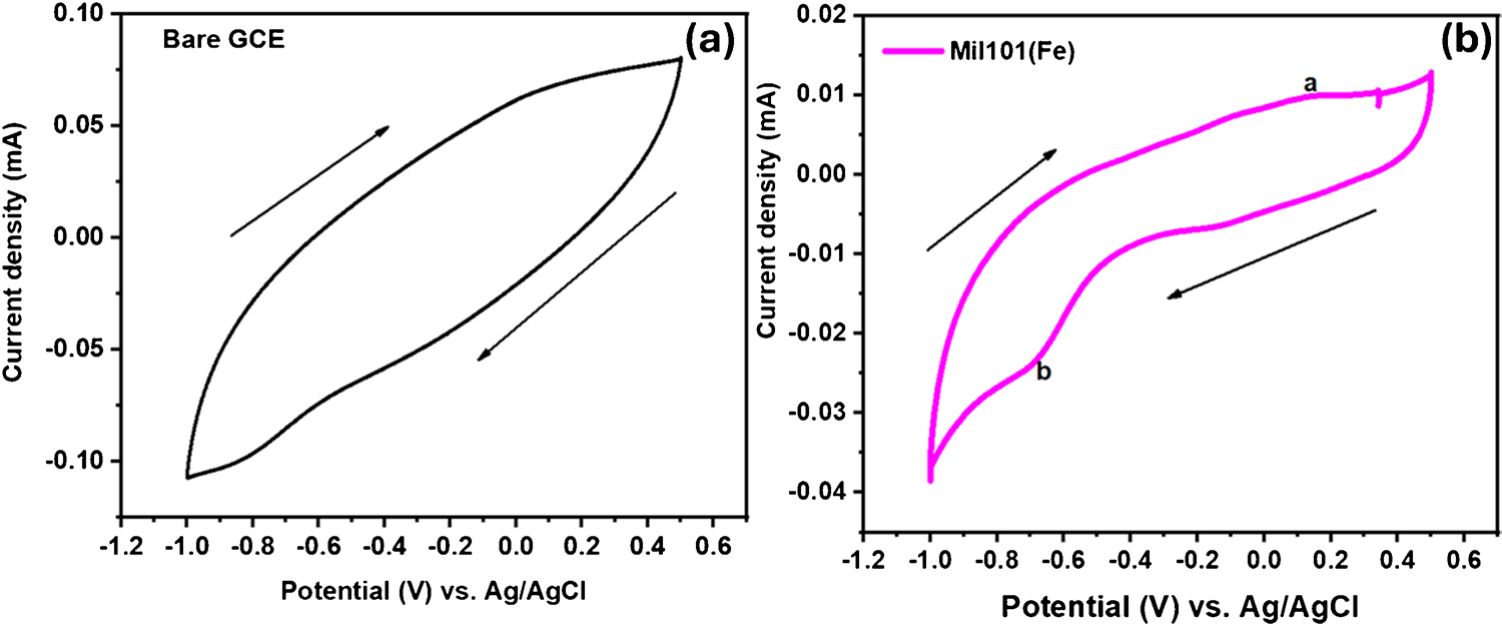
**a** The cyclic voltammograms of the bare electrode; **b** cyclic voltammograms of GCE-Mil101(Fe)

Figure 7a,b depicts the unmodified GCE and GCE-Mil101(Fe) electrodes in 0.1 M phosphate buffer (pH 7.2). The bare electrode did not exhibit any redox properties, and it was used for comparison purposes. When the GCE was modified with Mil101(Fe), a pair of redox properties was observed. GCE-Mil101(Fe) exhibited an oxidation peak current (ip_a_) at a peak potential of 0.143 V and anodic peak current (ip_b_) at a peak potential of − 0.69 V. These observations are attributed to surface redox reaction between Fe^3+^ and Fe^2+^, indicating its faradic behaviour [103].

**Fig. 8:**
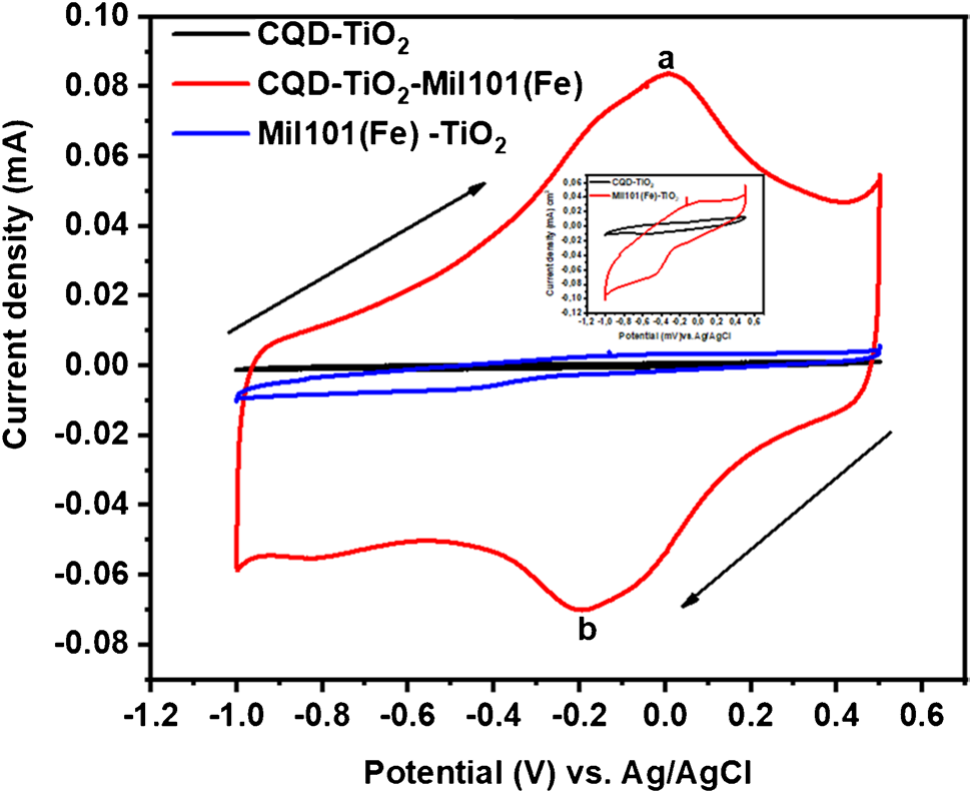
The cyclic voltammograms of a GCE-CQD-TiO_2_, GCE-Mil101(Fe)-TiO_2_, and GCE-Mil101(Fe)-CQD-TiO_2_ at a scan rate 50 mV s.^−1^, in phosphate buffer solution (pH 7.2)

The electrochemical behavior of the modified electrodes GCE-CQD-TiO_2_, GCE-Mil101(Fe)-TiO_2_, and GCE-Mil101(Fe)-CQD-TiO_2_ ternary composite was investigated in phosphate buffer solution at a pH of 7.2, using a sweep rate of 50 mV s^−1^, as shown in Fig. 8. The GCE-CQD-TiO_2_ ternary composite electrode exhibited oxidation and reduction peaks at potentials of − 0.0161 V and − 0.62 V, respectively, resulting in a peak-to-peak separation (Δ*E*_*p*_) of 0.624 V. In comparison, the GCE-Mil101(Fe)-CQD-TiO_2_ ternary composite electrode exhibited a higher current response than the GCE-CQD-TiO_2_, with oxidation and reduction peaks observed at 0.029 V and 0.488 V, respectively, resulting in a peak separation of 0.517 V. The GCE-Mil101(Fe)-CQD-TiO_2_ exhibited a well-defined redox pair. The modification of the ternary composite significantly increased the current response, with an oxidation peak (ip_a_) observed at a peak potential of 0.0174 V and a reduction peak (ip_b_) at a peak potential of − 0.213 V, resulting in a peak separation of 0.0230 V. The GCE modified with Mil101(Fe)-CQD-TiO_2_ showed a higher current response compared to both GCE-CQD-TiO_2_ and GCE-Mil101(Fe)-TiO_2_, along with a decreased Δ*E*_*p*_. This increase in current response and reduction in Δ*E*_*p*_ indicate that the GCE-Mil101(Fe)-CQD-TiO_2_ facilitates fast electron transfer kinetics between the electrode and the electrolyte. This improvement can be attributed to the properties of the ternary composite, which include a larger surface area and enhanced electrical conductivity. The ternary composite was used as an electrode modifier in further experiments.

**Fig. 9:**
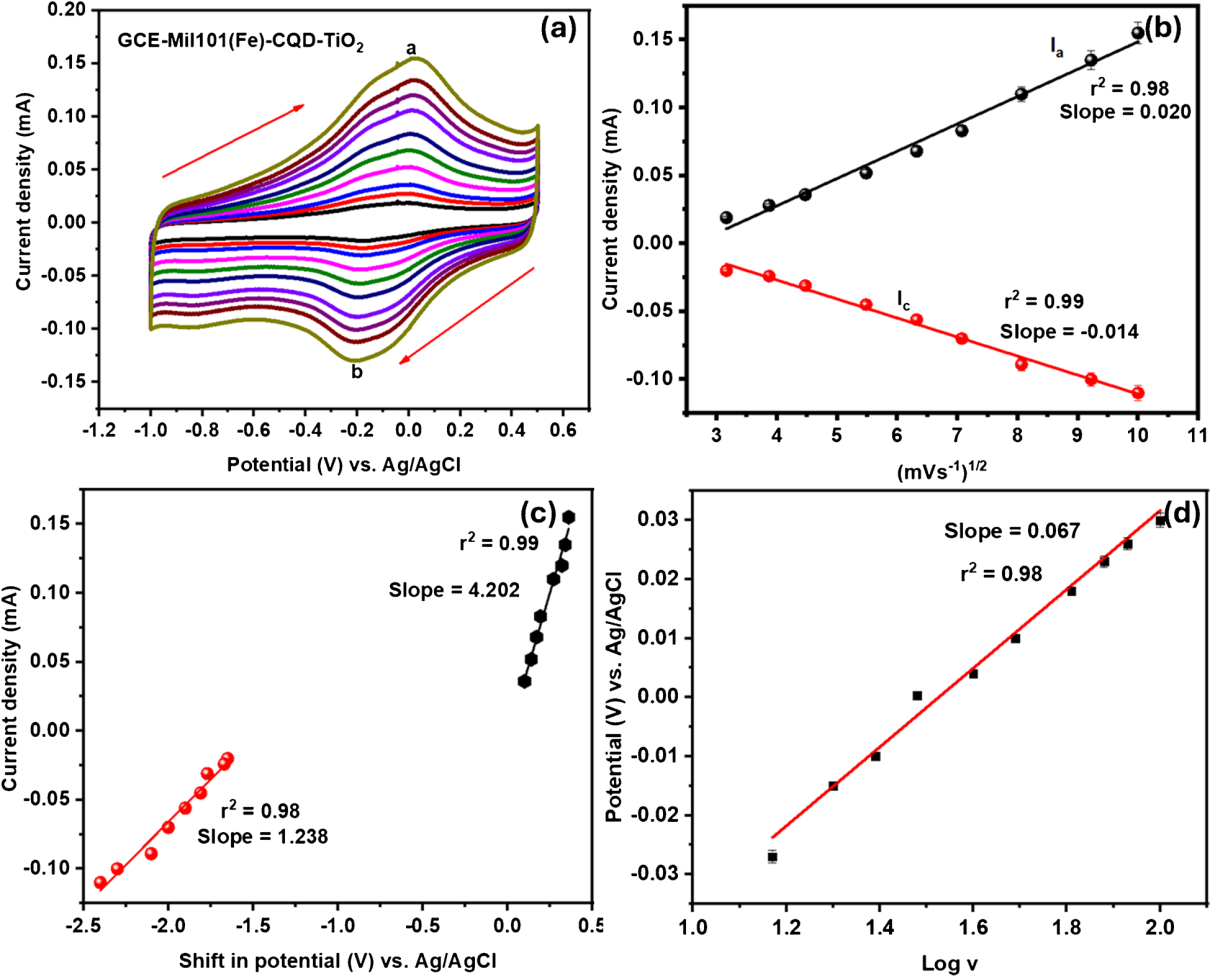
**a** The cyclic voltammograms of GCE-Mil101(Fe)-CQD-TiO_2_, **b** the linear plot of square root vs. current, **c** the shift potential vs. current, and **d** the plot of potential vs. log *v*

CV was used to analyze the anodic and cathodic peak characteristics by conducting different scan rates to determine the diffusion coefficient, adsorption–desorption processes, and electron mobility. GCE-CQD-TiO_2_-Mil101(Fe) exhibited an oxidation peak current (ip_a_) at a peak potential of 0.0174 V and a reduction peak current (ip_b_) at a peak potential of − 0.213 V. This observation of the redox peaks corresponds to the interaction between the Mil101(Fe), CQD, and TiO_2._ The -OH groups present in TiO_2_ create hydrogen bonds with the organic linkers in Mil101(Fe), resulting in a stable bond [104]. The Δ*E*_*p*_ for GCE-Mil101(Fe)-CQD-TiO_2_ was calculated to be 0.230 V, denoting that the system is quasi-reversible, as shown in Fig. 9a. The peak current is directly proportional to the square root of the scan rate, with correlation coefficients *r*^2^ = 0.98 and 0.99, as shown by the linear curve in Fig. 9b. The increase in peak current with the square root of the scan rate demonstrates the stability of the GCE-Mil101(Fe)-CQD-TiO_2_ and reveals a diffusion-controlled electrocatalytic process occurring at the electrode interface. Additionally, a slight shift in peak potential was observed with an increased scan rate, as illustrated in Fig. 9c. This suggests an irreversible electrochemical reaction process, supported by correlation coefficients (*r*^2^ = 0.99 and 0.98) [105]. Figure 9d shows the linear plot of log *v* vs. potential with a slope of 0.062 and *r*^2^ = 0.98. The diffusion coefficient and surface coverage were determined using the following equations: For quasi-reversible systems, the Randles–Sevcik and Laviron equations were employed [106, 107]:

1 $${I}_{p}=2.99x{10}^{5}na{(n\alpha )}^\frac{3}{2}{AD}^\frac{1}{2}{V}^\frac{1}{2} C$$

2 $${I}_{p}= \frac{{n}^{2}{F}^{2}AV\Gamma }{4RT}$$

where *I*_*p*_ is the peak current in amperes, *n*_*a*_ is the number of electron transfers, *D* is the diffusion coefficient (in cm^2^ s^−1^), *V* is the potential scan rate (scan rate in mV s^−1^), *C* is the concentration (in mol cm^−3^), α is the transfer coefficient, and *A* is the area of the electrode (in cm^2^) where *R* is the universal gas constant (8.3145 J mol^−1^), *T* is the temperature (298 K), and *F* is the Faraday constant 96,485 (in C mol^−1^), where Г is the surface coverage (in mol cm^−2^). To confirm and facilitate electron transport in the system, the diffusion coefficient (*D*) and surface coverage (Γ) were determined. The diffusion coefficient was calculated to be 2.576 × 10^−11^ cm^2^ s^−1^ and Г = 2.0609 × 10^−9^ mol cm^2^.

EIS is an electrochemical technique used to measure the impedance of an electrochemical system at various frequencies by applying a direct current voltage [108]. EIS works by perturbing the system while it is in a steady state or equilibrium. This method allows for the analysis of interactions at the electrode, such as charge transfer, concentration of electroactive species, and mass transfer from the bulk solution to the electrode surface [109]. The EIS technique was employed to investigate the diffusion mechanisms and the electron mobility at the electrode–electrolyte interface.

**Fig. 10:**
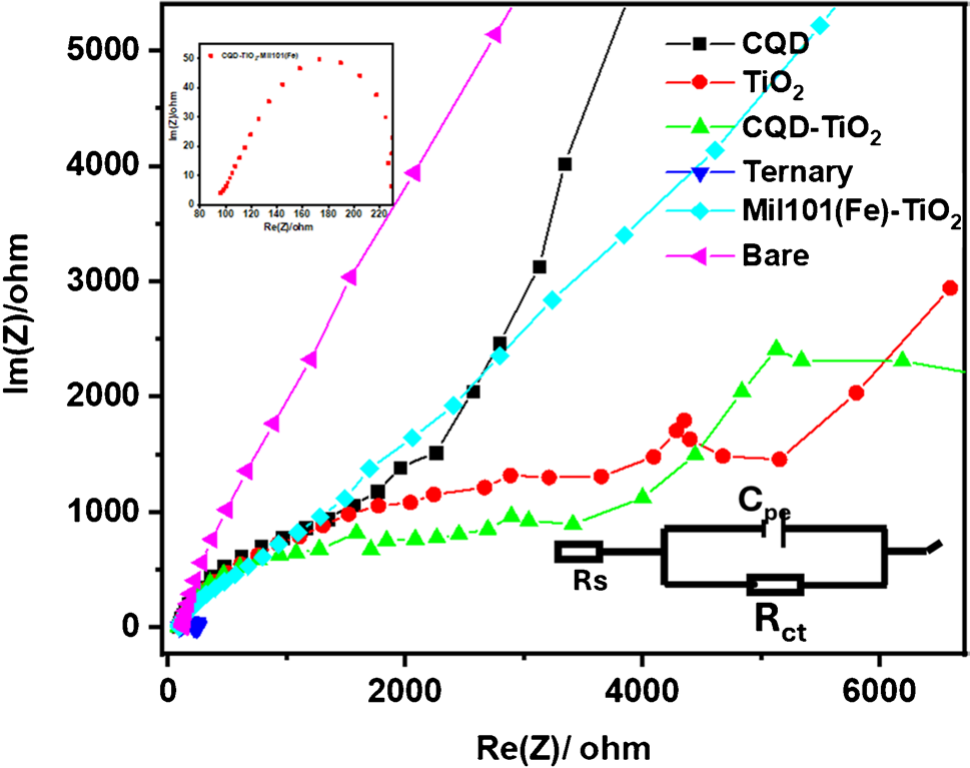
The Nyquist plot of GCE-CQD, GCE-TiO_2_, GCE-CQD-TiO_2_, GCE-Mil101(Fe)-TiO_2_, and GCE-Mil101(Fe)-CQD-TiO_2_

Figure 10 shows the Nyquist plots of GCE-CQD, GCE-TiO_2_, GCE-CQD-TiO_2_-Mil101(Fe), GCE-Mil101(Fe)-TiO_2_, and GCE. EIS experiments were conducted in phosphate-buffered saline at pH 7.2 to examine the kinetics of charge transfer of the modified electrodes. The EIS experiments were performed at frequencies ranging from 200 kHz to 100 mHz for all modified electrodes. The diameter of the semicircle provides the electron-transfer resistance (*R*_ct_) at the electrode–electrolyte interface, which evaluates the electrochemical performance of the system [110]. Rundle’s equivalent circuit was employed to interpret parameters such as the charge transfer resistance. The Mil101(Fe)-CQD-TiO_2_ modified electrode exhibits a lower practical *R*_ct_ compared to the GCE-CQD, GCE-TiO_2_, GCE-CQD-TiO_2_, and GCE electrodes, as well as the Mil101(Fe)-TiO_2_ modified electrode. The CQD-TiO_2_-Mil101(Fe) electrode exhibits a smaller diameter in the impedance arc, showing an *R*_ct_ of 36.87 Ω. In contrast, the GCE-TiO_2_ has an *R*_ct_ of 54.05 Ω, while the GCE-CQD shows an *R*_ct_ of 82.83 Ω, GCE-CQD-TiO_2_ with an *R*_ct_ of 45.91 Ω, GCE with an *R*_ct_ of 90 Ω, and GCE-Mil101(Fe)-TiO_2_ with an *R*_ct_ value of 39.49 Ω. These observations suggest that GCE-Mil101(Fe)-CQD-TiO_2_ facilitates higher electron transfer kinetics. Table 3 summarizes the electrochemical properties exhibited by the modified electrodes.

**Table 3:** Summary of the electrochemical properties demonstrated by the modified electrodes in 0.1 M PBS

Electrode	*R*_ct_ (Ω)	Δ*E*_*p*_	Diffusion controlled	Rs (Ω)	Synergy observed
GCE-CQD	82.83	0.61	No	81.88	NO
GCE-TiO_2_	54.05	0.81	No	69.45	NO
GCE-CQD-TiO_2_	45.91	0.559	Yes	94.59	Limited
GCE-Mof-TiO_2_	39.49	0.517	Yes	95.55	Moderate
GCE-Mil101(Fe)-CQD-TiO_2_	36.87	0.230	Yes	96.08	Strong synergistic effect

### Electrochemical response of the aptasensor

#### Optimization studies

**Fig. 11:**
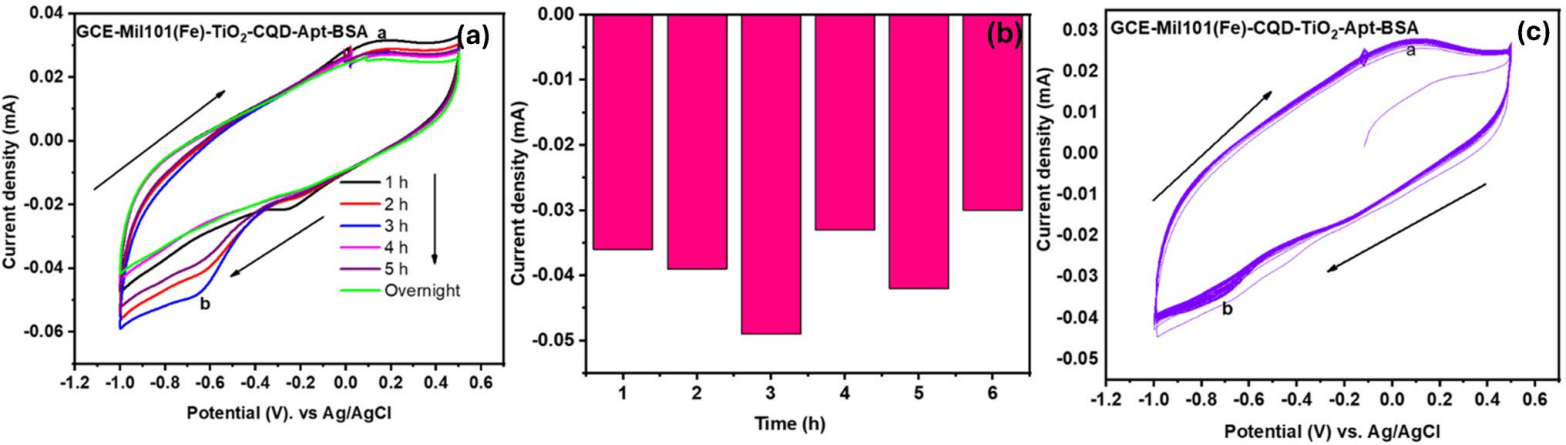
**a** SWV responses of GCE-Mil101(Fe)-CQD-TiO_2_-Apt-BSA, **b** inhibition binding curve of Crypto spiked concentration vs. current density, and **c** linear curve of Crypto spiked concentration vs. current density

**Fig. 12:**
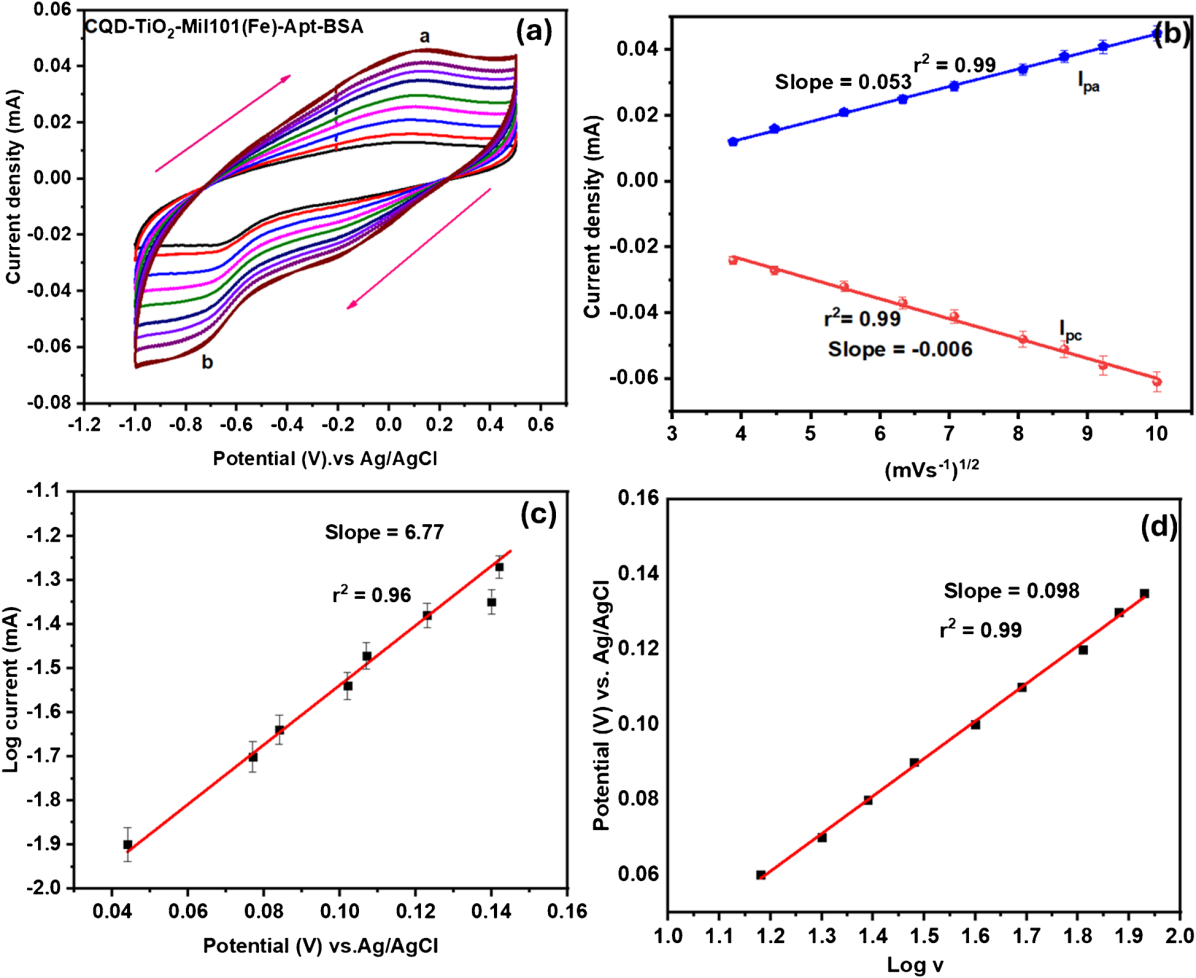
**a** Cyclic voltammograms of electrode incubation at different incubation periods, **b** inhibition curve of current density obtained at different incubation periods, and **c** cyclic voltammograms of GCE-CQD-TiO_2_-Mil101(Fe)-Apt-BSA

The developed GCE-CQD-TiO_2_-Apt-BSA was optimized by examining different incubation periods for the self-assembly of the aptamer layer on the working electrode. The working electrode was incubated for 1 h, 2 h, 3 h, 4 h, 5 h, and overnight in a solution containing the catalyst (Mil101(Fe)-CQD-TiO_2_) cross-linkers (EDC, NHS), binding buffer (tris buffer), aptamer solution, and a blocking agent (BSA) to optimize the aptasensor response. It can be observed from Fig. 11a that increasing the incubation time from 1 to 3 h significantly enhanced the reduction peak current (ip_b_) of the GCE-Mil101(Fe)-CQD-TiO_2_-Apta-BSA. However, extending the incubation time to overnight led to a decreased response of the aptasensor. These observations indicate that a 3-h incubation period effectively saturates all available active sites on the working electrode surface. Figure 12b illustrates the current response of the aptasensor at different incubation periods. The stability of the developed aptasensor was evaluated over time by conducting 100 cycles at a scan rate of 50 mV s^−1^, as shown in Fig. 12c. This parameter is essential for assessing the stability and reliability of electrochemical biosensors. The performance of the aptasensor was evaluated both before and after the stability test. Before the test, the aptasensor exhibited a current response of 0.0047 mA. In the first cycle of the stability test, the current response was 0.0025 mA, and by the 100th cycle, it had increased slightly to 0.0030 mA. Overall, the aptasensor’s performance decreased by 25% during the stability test.

**Fig. 13:**
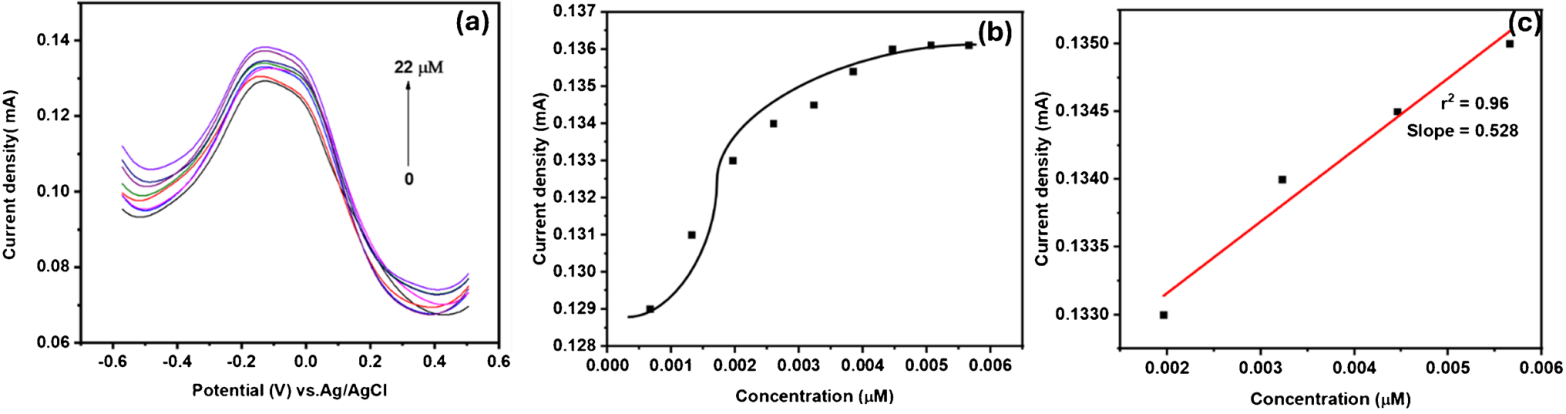
**a** The cyclic voltammograms of GCE-Mil101(Fe)-CQD-TiO_2_-Apt-BSA, **b** linear plot of square root vs. current, **c** potential vs. log current, and **d** log *v* vs. potential

CV experiments were performed within a potential range of 0.5 to − 1 V at various scan rates. The aim was to investigate the electrochemical properties, as well as the adsorption–desorption processes and diffusion coefficients, to determine the surface coverage, diffusion coefficients, and electroactive surface at the interface of the developed aptasensor. The platform exhibited an oxidation–reduction redox couple with a potential difference of 1.3 V, indicating that the aptasensor is quasi-irreversible, as shown in Fig. 13a [112]. The aptasensor exhibited an oxidation peak current at a potential (ip_a_) of 0.092 V, along with a reduction peak (ip_b_) current at a potential of − 0.79 V. This behavior indicates a potential electrostatic interaction between the active sites in the composite, specifically the carboxyl (-COOH) groups, and the amine bonds from the aptamer sequence. This interaction likely facilitates the formation of stable amide bonds [112]. An increase in scan rate with peak current was observed. These results indicate that both cathodic and anodic peak currents vary linearly with the square root of the scan rate, with correlation coefficients *r*^2^ of 0.99, as shown in Fig. 13b. This observation denotes that the system is undergoing a diffusion-controlled charge transfer electrocatalytic reaction. Figure 13c illustrates the Tafel plot of log current against potential. Tafel slope measures how effectively an electrode can generate current in response to potential [113]. Additionally, the Tafel slope is used to evaluate the rates and mechanisms of electrocatalytic reactions. To further confirm that the aptasensor is a quasi-reversible system, the transfer coefficient (α) was calculated using the slope of Fig. 13d. The values of the current and potential used in this plot were taken from the oxidation peaks of Fig. 13a [115]. The slope of this plot was used to determine the transfer coefficient using the following equation:

3 $$\text{Slope}= \frac{\alpha F}{2.303 \times \text{RT}}$$

where *F* is Faraday’s constant 96,485 C mol^−1^, *R* is the universal gas constant (8.314 J mol^−1^), and *T* is the temperature (298 K). The calculated α was determined to be 0.40, which lies in the quasi-reversible zone [115]. The kinetic constant was determined using the following equation. The calculated *K*^o^ is equal to 6.72 × 10^−5^ cm^−1^, indicating slow electron movement between the electrode–electrolyte interface and the electroactive species. This value is comparable to the literature and confirms a quasi-reversible system [113, 116].

4 $$\text{ln}\left({k}^{o}\right)= \frac{\alpha nF}{\text{RT}}\left({E}_{{p}^{-}}{E}^{o}\right)-\text{ln} (\frac{\text{RT}}{\alpha nF})$$

Figure 13d shows the Tafel plot of log scan rate against potential. The Tafel slope was determined by plotting the oxidation peak potential versus the log of the scan rate. The slope of this plot was used in the following equation, where *b* is the Tafel slope and *K* is the kinetic constant. Tafel slopes between 60 and 120 mV/decade are particularly advantageous, as they imply one electron transfer during the rate-determining step. The calculated Tafel slope was equal to 49 mV/decade.

The Tafel slope was determined to be

5 $${E}_{p = }\frac{b}{2}\text{ log}v+k$$

The diffusion coefficient and surface coverage were determined using the Randles–Sevcik and Laviron equations to confirm electron mobility. The calculated diffusion coefficient (*D*) is 5.994 × 10^−11^ cm^2^ s^−1^, while the surface coverage (Γ) is 6.747 × 10^−9^ mol cm^−2^. The surface coverage of the aptasensor increased from 2.0609 × 10^−9^ mol cm^−2^ for the Mil101(Fe)-CQD-TiO₂ to 6.747 × 10^−9^ mol cm^−2^. To evaluate the contribution of the aptamer, a control experiment was conducted using a glassy carbon electrode modified with bovine serum albumin (GCE-BSA). The surface coverage obtained for this electrode was 1.199 × 10^−9^ mol cm^−2^. By subtracting the surface coverage of GCE-BSA from that of the GCE-Mil101(Fe)-CQD-TiO₂-Apt-BSA, the adjusted surface coverage (Γ) value becomes 5.557 × 10^−9^ mol cm^−2^.

The slope value of peak current vs. the square root of the scan rate plot (Fig. 13b) was used to calculate the electroactive surface area (EASA) of the GCE-Mil101(Fe)-CQD-TiO_2_ Apt-BSA using the Randles–Sevcik equation.

$${I}_{p}=2.99x{10}^{5}n{\left(na\right)}^\frac{3}{2}{AD}^\frac{1}{2}{V}^\frac{1}{2} C$$

$$\text{EASA}=\frac{m}{\left(2.99\times 1{0}^{5}\right)\times {n}_{2}^{3}\times A\times {D}_{2}^{1}\times C}$$

where *m* is the slope value, *n* is the number of electron transfers, *A* is the area of the electrode (in cm^2^), *D* is the diffusion coefficient (in cm^2^ s^−1^), and *C* is the concentration (in mol cm^−3^). The calculated EASA was 3.224 cm^2^. This indicates that the GCE-Mil101(Fe) CQD-TiO₂-Apt-BSA has more active sites for analyte detection.

#### Concentration-dependent studies

The relationship between the spiked Crypto concentrations, ranging from 3 to 22 µM, and oxidation peak currents was studied using the SWV technique in phosphate buffer solutions (pH 7.2) at a sweep rate of 50 mV s^−1^ to examine the performance of the developed aptasensor. Figure 11a–c depicts the SWV response on spiked Crypto concentrations, Fig. 11b shows the binding inhibition curve of spiked Crypto concentration against current density, and Fig. 11c illustrates the linear plot of spiked Crypto concentration vs. current density. Figure 11a illustrates the SWV response at various spiked concentrations of Crypto. An increase in oxidation peak current was observed with an increase in the concentration of Crypto. This observation indicates the binding affinity of Crypto on the surface of GCE-Mil101(Fe)-CQD-TiO_2_-Apt-BSA. Figure 11b shows the inhibition curve of spiked Crypto concentrations vs. current density. It was observed that after spiking with a 9 µM concentration, the platform reached a saturation point. The relationship between the Crypto concentration and current density is illustrated by a linear curve in Fig. 11c, with a correlation coefficient of *r*^2^ = 0.96 and a slope of 0.528. The limit of detection of the aptasensor was calculated using the following equation.

$$\text{LOD }\frac{3\partial }{m}$$

where m is the slope of the linear curve of spiked concentration vs. current response and 3 (ꝺ) is the standard deviation (*n* = 10). The calculated standard deviation was 0.0002. The aptasensor exhibited a LOD of 0.001 µM, a sensitivity of 0.529 mA/µM, and a linear range of 0.0015–0.004 µM.

**Fig. 14:**
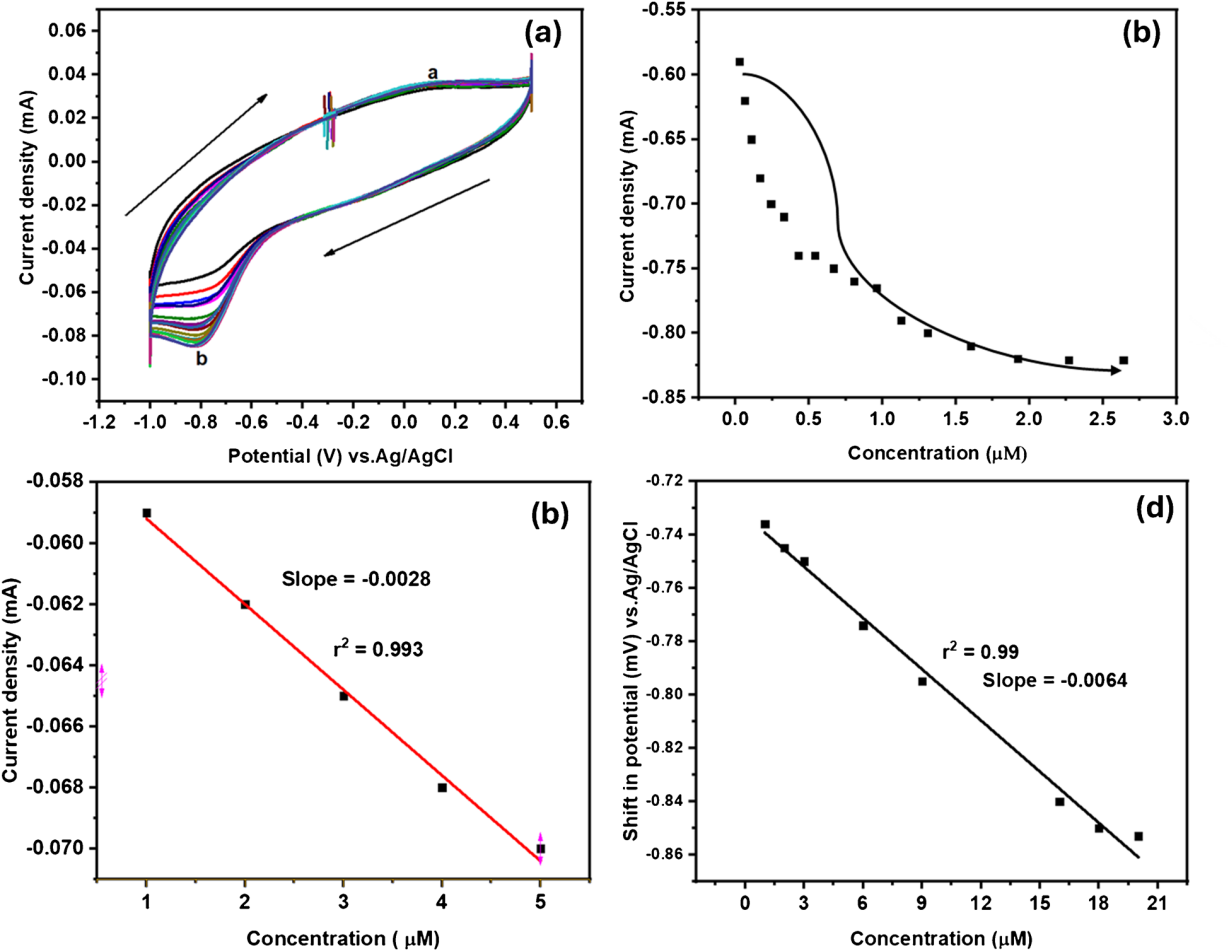
**a** The CV response on spiked Crypto concentration, **b** inhibition binding curve of spiked Crypto concentrations vs. current density, **c** spiked Crypto concentration vs. current, and **d** linear curve of shift in potential vs. spiked Crypto concentrations

Cyclic voltammetry was conducted to examine the behavior of oxidation–reduction of GCE-Mil101(Fe)-CQD-TiO_2_-Apt-BSA in the presence of spiked Crypto concentrations ranging from 3 to 22 µM, using a sweep scan of 50 mV s^−1^, PBS (pH 7.2). Figure 14a–d illustrates the cyclic voltammograms in the presence of spiked Crypto concentration, Fig. 14b the inhibition curve of spiked Crypto concentration vs. current density, Fig. 14c the linear plot of spiked Crypto concentration vs. current density, and Fig. 14d the spiked Crypto concentrations vs. shift in potential. Figure 14a shows the cyclic voltammetry response to Crypto concentrations. The interaction between the aptasensor and the spiked Crypto concentration resulted in an increase in the ip_b_ of GCE-Mil101(Fe)-CQD-TiO_2_-Apt-BSA. This observation suggests that the developed sensing platform exhibits the electrochemical characteristics for the electro-reduction of Crypto. Figure 14b depicts the binding inhibition curve of spiked Crypto concentration against the current response. The correlation between the current response and spiked Crypto concentrations is illustrated by a linear curve in Fig. 14c with a slope of − 0.0028 and a correlation coefficient of *r*^2^ = 0.993. The increase in reduction peak of GCE-Mil101(Fe)-CQD-TiO_2_-Apt-BSA with an increase in spiked Crypto concentrations resulted in a slight shift in peak potential, denoting fast electron transfer kinetics at the electrode electrolyte interface, as illustrated by Fig. 14d with *r*^2^ = 0.99.

**Fig. 15:**
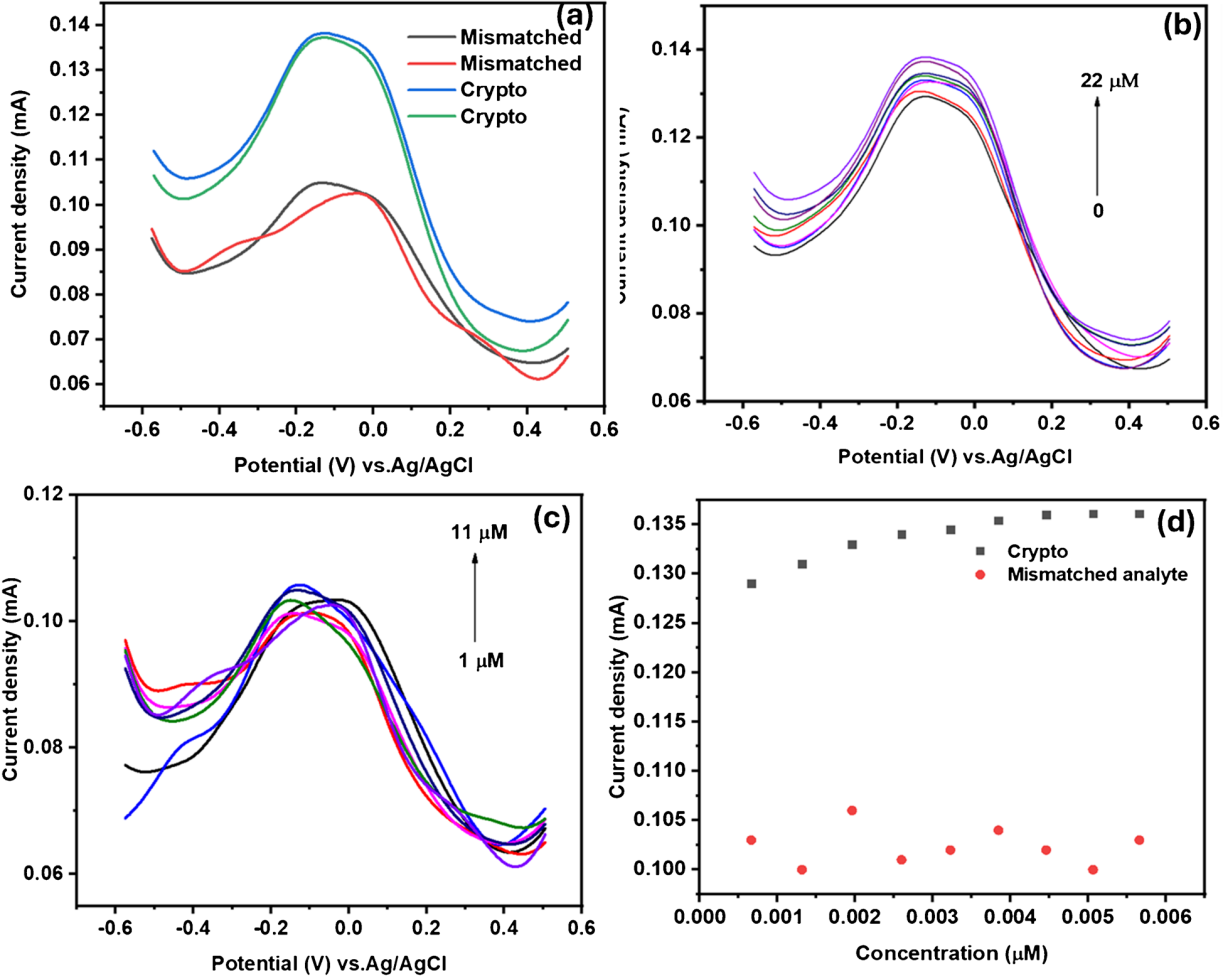
**a** The current response for Crypto detection compared to that of mismatch analyte, **b** the SWV response of GCE-Mil101(Fe)-CQD-TiO_2_-Apt-BSA at different spiked Crypto concentrations, and **c** the SWV response of GCE-Mil101(Fe)-CQD-TiO_2_-Apt-BSA at various mismatched analyte concentrations. **d** The binding curves obtained for Crypto and mismatched analyte detections

The selectivity of the developed aptasensor was assessed by detecting a mismatched analyte. The experiment was conducted in a window of 0.5–1 V using phosphate buffer solution (pH 7.2) as an electrolyte at a scan rate of 50 mV s^−1^. The prepared concentrations of the analyte ranged from 1 to 11 µM. Figure 15a–d compares the SWV response of GCE-Mil101(Fe)-CQD-TiO_2_-Apt-BSA for the detection of Crypto with that of the mismatched analyte. Figure 15b shows the SVW response of GCE-Mil101(Fe)-CQD-TiO_2_-Apt-BSA at varying spiked Crypto concentrations. Figure 15c illustrates the SWV response of GCE-Mil101(Fe)-CQD-TiO_2_-Apt-BSA to varying spiked mismatched analytes, and Fig. 15d represents the two linear curves obtained from Crypto detection and mismatched analyte detection. As shown in Fig. 15a, the SWV response of Crypto detection exhibited a higher current response compared to the mismatched analyte. Figure 15b depicts the SWV response to the spiked Crypto concentration. It was observed that the current response increased with an increase in spiked Crypto concentrations. Indicating a good correlation between the platform and Crypto detection. Figure 15c shows the SWV response on various mismatch analyte concentrations. There was no binding affinity between the developed aptasensor and the spiked concentrations. This suggests that a repulsion process was occurring at the electrode–electrolyte interface, as illustrated by the inhibition binding curve in Fig. 15d.

There is limited work done on the detection of Crypto using electrochemical sensors. The aptasensor developed in this study is compared with other electrochemical sensors developed for detecting *Cryptosporidium* in water and buffer samples, as illustrated in Table 4.

**Table 4:** Comparison of electrochemical sensors used for the detection of *Cryptosporidium* with this work

Modified electrodes	Technique	Limit of detection (LOD)	Linear range	References
Au-SPCE	EIS	0.003 ng/mL	0.005–0.02 ng/mL	[117]
GNPs-SPCE	SWV	0.01 ng/mL	0.015–0.08 ng/mL	[118]
ITO-AuNPs	DPV	0.003 ng/mL	0.003–0.005 ng/mL	[119]
AuNPs-SPCE-Apt	EIS	0.010 ng/mL	0.05–0.02 ng/mL	[120]
Mil101(Fe)-CQD-TiO_2_-Apt-BSA	SWV	0.001 ng/mL	0.001–0.004 ng/mL	This work

**Fig. 16:**
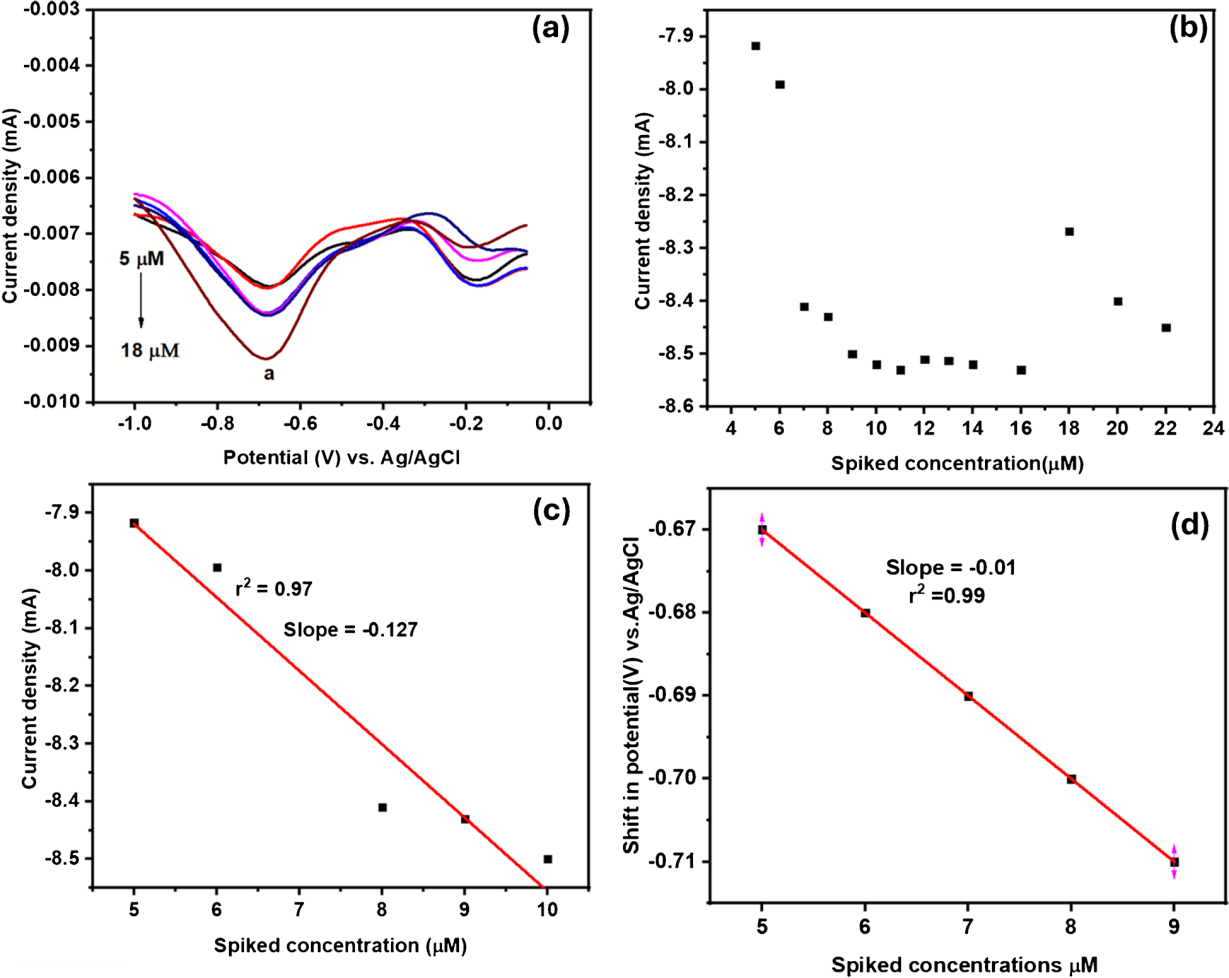
**a** The SWV experiments in the presence of spiked cadmium ion concentrations, **b** the binding inhibition curve, **c** the spiked cadmium ion concentrations vs. current response, and **d** the shift in potential vs. spiked concentrations

**Fig. 17:**
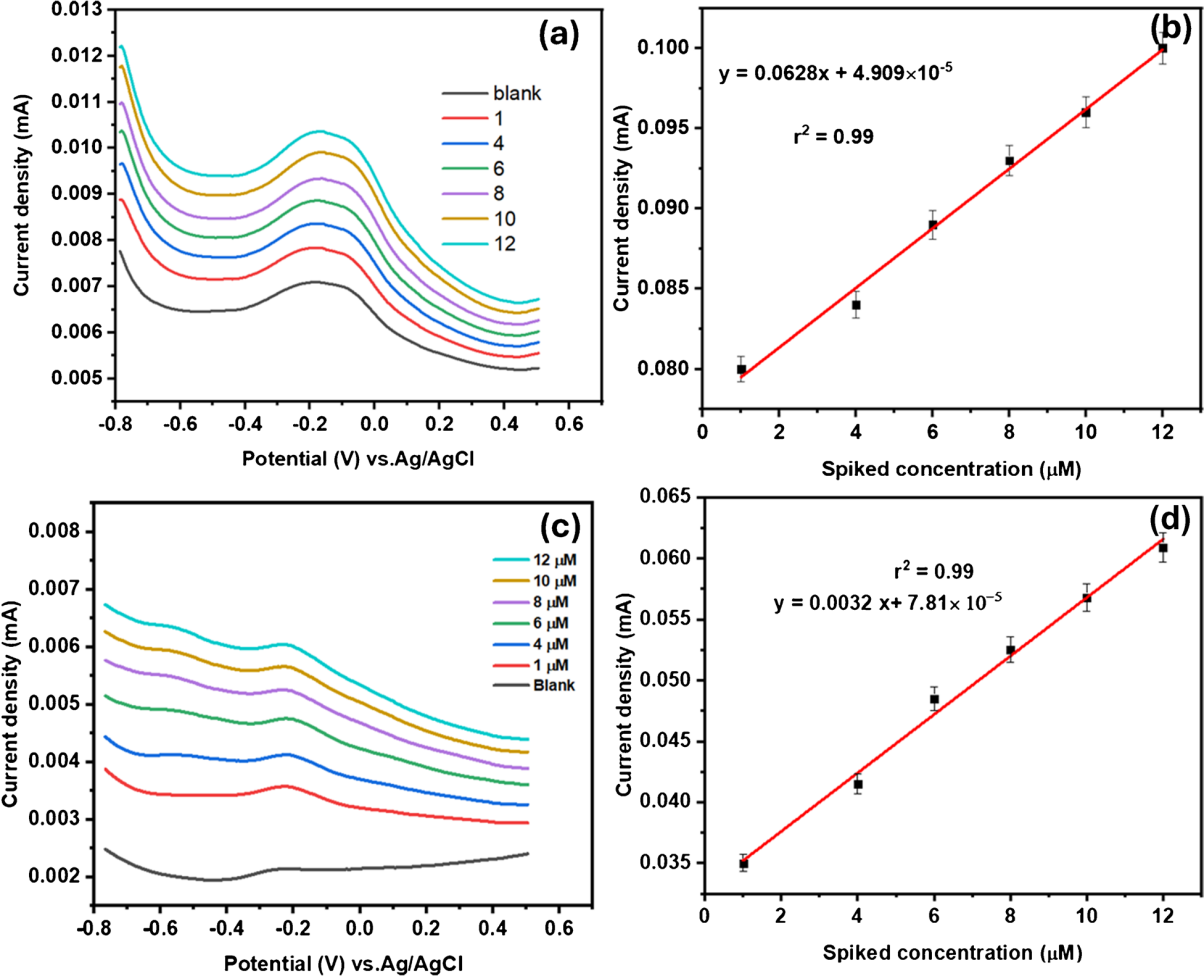
**a** The SWV response to varying spiked Cd^2+^ concentration in wastewater, **b** linear curve of spiked concentration vs. peak current, **c** the SWV experiment to different Cd^2+^ spiked concentrations, and **d** the linear curve of current vs. spiked concentrations in tap water

The behavior of the developed aptasensor was also examined using the SWV technique to detect cadmium ions in buffer solutions. The experiments were conducted at room temperature using a potential window of 0.5 V to − 1 V at a scan rate of 50 mV s^−1^ in a phosphate buffer solution (pH 7.2). The reduction peak was chosen for the cadmium ion SWV experiments because it displayed a well-defined peak and demonstrated a higher current response compared to the oxidation peaks. This observation was attributed to the quasi-reversible nature of the redox couple. Figure 16a–d shows the SWV response on spiked cadmium concentrations, Fig. 16b the inhibition binding curve of cadmium ions on GCE-Mil101(Fe)-CQD-TiO_2_-Apt-BSA surface, Fig. 16c the spiked cadmium ions vs. current density, and Fig. 16d the shift in potential vs. current density. Figure 17a illustrates the results of SWV experiments conducted with varying concentrations of spiked cadmium ions. As the concentration of cadmium ions increased, there was a corresponding rise in the SWV reverse scan. This observation indicates that cadmium ions have a binding affinity for the surface of the developed aptasensor. The aptasensor successfully detected cadmium ions up to a concentration of 9 µM at a peak potential of 0.68 V (ip_a_). These results are consistent with the literature [118, 119]. The aptasensor reached a point of saturation after the addition of 10 µM, demonstrating a linear range of 10–16 µM. Figure 16b shows the binding inhibition curve of cadmium ions on GCE-Mil101(Fe)-CQD-TiO_2_-Apt-BSA surface. Figure 16c illustrates the correlation between the spiked cadmium concentrations vs. the current response with a correlation coefficient of *r*^2^ = 0.97 and a slope of 0.217. As cadmium ion concentrations increased in the interaction with the developed aptasensor, a slight shift in peak potential was observed, indicating rapid electron transfer at the aptasensor surface. Figure 16d illustrates the linear relationship between the shift potential and the current response, with a correlation coefficient of *r*^2^ = 0.99. The results suggest that the developed GCE-Mil101(Fe)-CQD-TiO_2_-Apt-BSA is a multifunctional system capable of detecting a range of pollutants. The LOD was determined using the following equation:

$$\text{LOD} \frac{3\partial }{m}$$

The aptasensor exhibited a LOD of 0.005 µM with a sensitivity of 0.127 mA/µM. Comparison of the electrochemical sensors used for cadmium ion detection with the developed aptasensor is shown in Table 5.

**Table 5:** Comparison of electrochemical techniques used for detecting cadmium ions

Modified electrode	Technique	Limit of detection (LOD)	Linear range	References
N-NC-GCE	SWV	10.84 µM	1.46–73 µM	[121]
APTE-Mono@-GCE	DPV	7.37 µM	4–80 µM	[122]
NiWO_4_-MWCNTs-GCE	SWV	0.12 µM	50–450 µM	[7]
Apt-MSH-GCE	SWV	0.0095 µM	0.001–0.05	[123]
SWCNT-GCE	SWV	9.44 µM	12–100 µM	[124]
ZIF-rGO-GCE	SWASV	10.11 µM	45–433 µM	[125]
AuNP-CD-GCE	DPV	0.99 µM	1–100 µM	[126]
SO-GCE	SWV	3.3 × 10^−11^ µM	1.0 × 10^−10^–5.0 × 10^−8^ µM	[126]
Mil101-CQD-TiO_2_-GCE	SWV	0.005 µM	7–11 µM	This work

## Real-water application

**Fig. 18:**
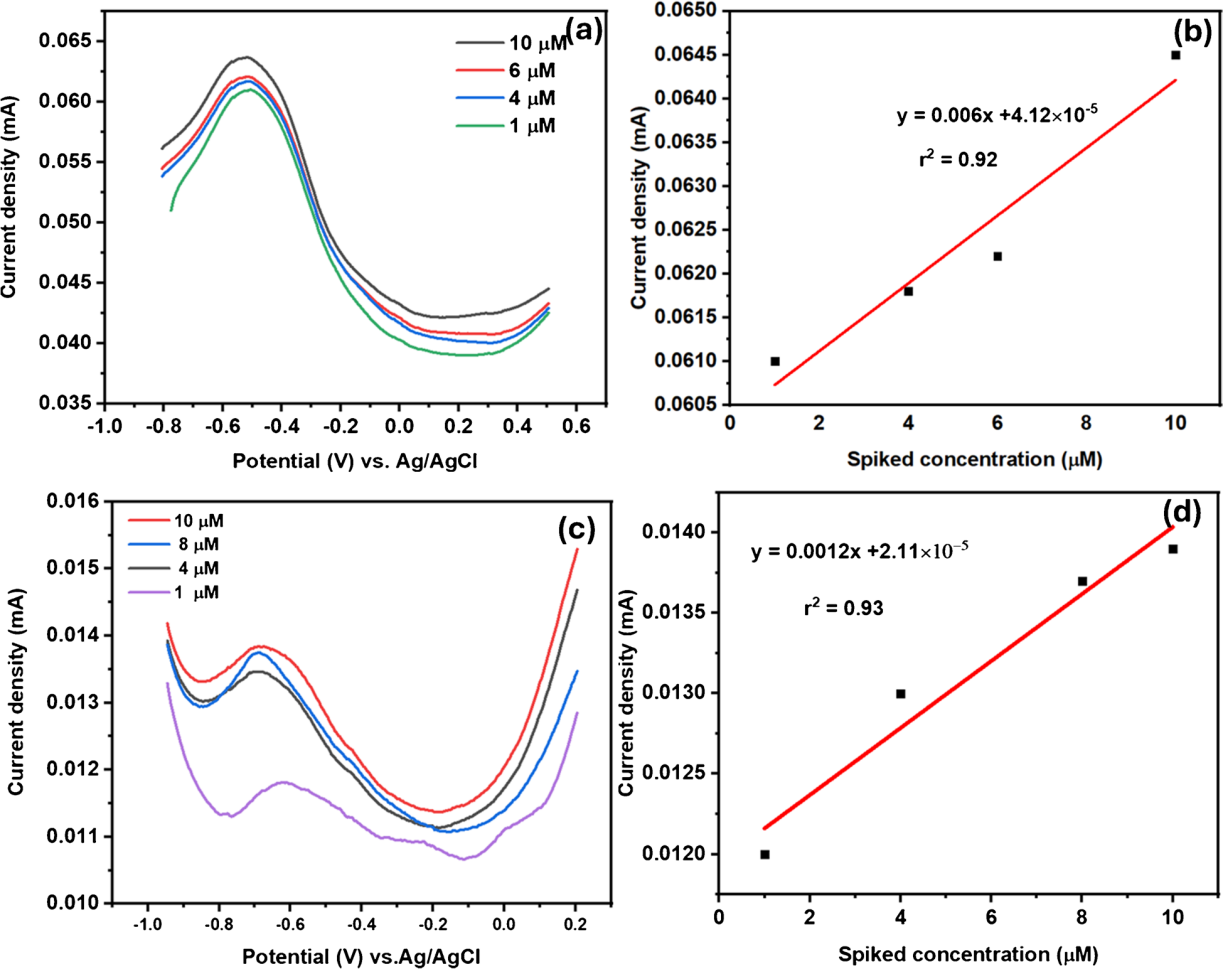
**a** The SWV response to varying spiked Crypto concentration in wastewater, **b** linear plot of spiked concentration vs. current response, **c** the SWV experiment to different spiked concentrations in tap water, and **d** the linear curve of current vs. spiked concentrations

The developed GCE-Mil101(Fe)-CQD-TiO_2_-Apt-BSA was evaluated in both wastewater and tap water through SWV experiments. These experiments were conducted over a potential window ranging from − 0.8 to 0.5 V, with a scan rate of 50 mV s^−1^. Prior to the analysis, the wastewater was filtered, and the pH of both the wastewater and tap water was adjusted to 7.2. During the SWV experiments, a blank measurement was taken using the collected water samples. After assessing the response of the aptasensor’s behavior, the working concentrations ranged from 1 to 12 µM for Crypto and 1 to 10 µM for Cd^2+^, which were spiked into water samples (wastewater and tap water). Figure 18a illustrates the SWV response to varying concentrations of spiked Crypto in wastewater. The current response increased linearly with increasing spiked concentration, yielding a correlation coefficient of *r*^2^ = 0.99 as depicted in Fig. 18b. Figure 18c shows the SVW response to different spiked Crypto concentrations in tap water. The aptasensor response in wastewater was notably higher than in tap water, as shown in Fig. 18c. Furthermore, the current also increased with an increase in spiked concentrations, as illustrated in Fig. 18d. The recoveries for the detection of Crypto in both water samples are shown in Table 6, ranging from 87 to 98%. For tap water, the recoveries ranged from 67 to 82%. Overall, the aptasensor demonstrated superior performance in wastewater samples compared to those in tap water. Figure 17a–d depicts the Cd^2+^ detection in real water samples. Figure 17a shows the SWV experiment in wastewater samples in the presence of spiked Cd^2+^. It was well noted that the peak current increased with an increase in spiked concentrations. The relationship between peak current and spiked concentrations is represented in Fig. 17b, with a correlation coefficient of (*r*^2^ = 0.92). Figure 17c shows the SWV response to varying concentrations of spiked Cd^2+^. The current response also increased linearly with the spiked Cd^2+^ concentrations, yielding a linear curve with an *r*^2^ value of 0.93. The aptasensor demonstrated a comparable response for Cd^2+^ in both wastewater and tap water samples, resulting in recoveries ranging from 72–89.2%. Table 7 summarizes the results obtained from Fig. 17a–d.

**Table 6:** Data obtained for the detection of Crypto in real water samples with recoveries

Sample	Added (µM)	Found (µM)	Recovery (%)
Wastewater	1	0.127	87.3
	4	0.140	96.5
	6	0.147	97.55
	8	0.152	98.1
Tap water	4	1.31	67.3
	6	1.5	75.77
	8	1.75	82.28

**Table 7:** The summarized results obtained for the detection of Cd^2+^ in real water samples

Sample	Added (µM)	Found (µM)	Recovery (%)
Wastewater	4	1.203	74.77
	6	1.03	82.20
	10	1.068	89.6
Tap water	4	1.083	72.92
	8	1.058	86.77
	10	1.15	88.54

## Summary and conclusion

The hydrothermal method was effectively used to synthesize a unique ternary nanocomposite based on Mil101(Fe)-CQD-TiO_2_, which was then extensively characterized by XRD, FTIR, HR-TEM, Raman spectroscopy, CV, and EIS. The consistent integration of titanium dioxide and carbon quantum dots onto the Mil101(Fe) framework was validated by the structural and morphological investigation, resulting in a high-surface-area composite with exceptional conductivity. According to electrochemical measurements, the ternary composite exhibited good monolayer aptamer immobilization (*ᴦ* = 6747 × 10^−9^ mol cm^−2^), a higher electroactive surface (3.22 cm^2^), and significantly increased electron transfer kinetics, all of which improved sensor performance. In the development of a label-free aptasensor, the composite-enabled platform showcased impressive analytical performance for both cadmium ions (Cd^2^⁺) and *Cryptosporidium parvum*. It exhibited high sensitivity, with a response of 0.528 mA/µM for *Cryptosporidium*, and a low detection limit of 0.001 µM for cadmium ions. For Cd^2^⁺, the sensitivity measured was 0.127 mA/µM, with a detection limit of 0.073 µM. The aptasensing platform demonstrated superior performance in real water samples, with acceptable recoveries ranging from 67 to 98% for *Cryptosporidium parvum* and 72 to 89% for Cd^2+^. The specificity of the aptasensor was demonstrated through selectivity studies, which showed a slight reaction to mismatched analytes. The findings position the platform as a flexible instrument for biological and environmental sensing and point to its wider usefulness for identifying other heavy metals and microbial diseases.

## Future perspectives

1. Real-time point-of-care prototype development (portable potentiostat and disposable sensors)
2. Miniaturization on paper-based platforms for cost-effective environmental diagnostics
3. Integration with microfluidics for autonomous sample processing and detection

## Data Availability

No datasets were generated or analysed during the current study.
